# The nucleosome remodeling and deacetylase-SWItch/sucrose non-fermentable antagonism regulates the coordinated activation of epithelial-to-mesenchymal transition and inflammation in oral cancer

**DOI:** 10.1093/jnci/djaf065

**Published:** 2025-03-20

**Authors:** Roberto Stabile, Francesco A Tucci, Mathijs P Verhagen, Carmen Embregts, Thierry P P van den Bosch, Rosalie Joosten, Maria J De Herdt, Berdine van der Steen, Alex L Nigg, Senada Koljenović, Jose A Hardillo, Cornelis Peter Verrijzer, Adrian Biddle, Robert J Baatenburg de Jong, Pieter J M Leenen, Riccardo Fodde

**Affiliations:** Department of Pathology, Erasmus University Medical Center, 3000 CA Rotterdam, The Netherlands; Department of Pathology, Erasmus University Medical Center, 3000 CA Rotterdam, The Netherlands; Department of Pathology, Erasmus University Medical Center, 3000 CA Rotterdam, The Netherlands; Department of Viroscience, Erasmus University Medical Center, 3000 CA Rotterdam, The Netherlands; Department of Pathology, Erasmus University Medical Center, 3000 CA Rotterdam, The Netherlands; Department of Pathology, Erasmus University Medical Center, 3000 CA Rotterdam, The Netherlands; Department of Otorhinolaryngology and Head & Neck Surgery, Erasmus University Medical Center, 3000 CA Rotterdam, The Netherlands; Department of Otorhinolaryngology and Head & Neck Surgery, Erasmus University Medical Center, 3000 CA Rotterdam, The Netherlands; Department of Pathology, Erasmus University Medical Center, 3000 CA Rotterdam, The Netherlands; Department of Pathology, Erasmus University Medical Center, 3000 CA Rotterdam, The Netherlands; Department of Otorhinolaryngology and Head & Neck Surgery, Erasmus University Medical Center, 3000 CA Rotterdam, The Netherlands; Department of Biochemistry, Erasmus University Medical Center, 3000 CA Rotterdam, The Netherlands; Centre for Cell Biology and Cutaneous Research, Blizard Institute, Queen Mary University of London, E1 2AT London, United Kingdom; Department of Otorhinolaryngology and Head & Neck Surgery, Erasmus University Medical Center, 3000 CA Rotterdam, The Netherlands; Department of Immunology, Erasmus University Medical Center, 3000 CA Rotterdam, The Netherlands; Department of Pathology, Erasmus University Medical Center, 3000 CA Rotterdam, The Netherlands

## Abstract

**Background:**

Phenotypic plasticity and inflammation, 2 well-established hallmarks of cancer, play key roles in local invasion and distant metastasis by enabling the rapid adaptation of tumor cells to dynamic micro-environmental changes.

**Results:**

Here, we show that in oral squamous carcinoma cell carcinoma (OSCC), the competition between the Nucleosome Remodeling and Deacetylase (NuRD) and SWItch/Sucrose Non-Fermentable (SWI/SNF) chromatin remodeling complexes plays a pivotal role in regulating both epithelial-mesenchymal plasticity (EMP) and inflammation. By perturbing these complexes, we demonstrated their opposing downstream effects on the inflammatory pathways and EMP regulation. In particular, downregulation of the BRG1-specific SWI/SNF complex deregulates key inflammatory genes, such as TNF-α and IL6, in opposite ways when compared with the loss of *CDK2AP1*, a key member of the NuRD complex. We showed that *CDK2AP1* genetic ablation triggers a pro-inflammatory secretome encompassing several chemokines and cytokines, thus promoting the recruitment of monocytes into the tumor microenvironment (TME). Furthermore, *CDK2AP1* deletion stimulates their differentiation into M2-like macrophages, as validated on tumor microarrays from OSCC patient-derived tumor samples. Further analysis of the inverse correlation between CDK2AP1 expression and TME immune infiltration revealed specific downstream effects on the abundance and localization of CD68^+^ macrophages.

**Conclusions:**

Our study sheds light on the role of chromatin remodeling complexes in OSCC locoregional invasion and highlights the potential of CDK2AP1 and other members of NuRD and SWI/SNF chromatin remodeling complexes as prognostic markers and therapeutic targets.

## Introduction

Oral squamous cell carcinoma (OSCC) is the most prevalent type of head and neck cancer, representing over 90% of malignancies originating from the mucosal epithelium of the oral cavity.^1^^,^^2^ OSCC is associated with various risk factors, including tobacco and alcohol use, as well as viral infections, such as human papillomavirus (HPV). However, unlike other sub-sites in the head and neck region, HPV is responsible for only a small percentage (2%-5%) of OSCC cases, and its significance in this context remains uncertain.^1^^,^^3^ Improving the prognosis of the HPV-negative patients presents an unmet need, as mortality outcomes have shown limited improvement over the past few decades, with less than 50% of this subset of OSCC patients surviving beyond 5 years.^4^

Conventional treatment modalities for OSCC, such as surgery and radiotherapy, have yielded suboptimal results and can lead to significant morbidity. Understanding the mechanisms underlying OSCC invasion and metastasis remains a major area of research as it accounts for the majority of cancer-related deaths, with more than 50% of patients experiencing cancer recurrence or developing metastases within 3 years of treatment.^5-7^

To navigate the complex cascade of events underlying metastasis, cancer cells must acquire the ability to dynamically adapt to varying environmental conditions through reversible alterations in cellular identity. This phenotypic plasticity is governed by epigenetic mechanisms that do not alter the genetic code but instead control how information encoded in DNA is expressed in a tissue- and context-specific manner.^6^^,^^8^ In particular, chromatin-remodeling complexes play critical roles in modulating gene expression programs that are essential for cellular homeostasis and development. These complexes facilitate dynamic changes in chromatin structure, allowing precise regulation of gene transcription. Deregulation of chromatin remodeling is now considered a hallmark of cancer and contributes to the transient changes in gene expression patterns observed in tumor cells en route to their metastatic destinations.^6^^,^^9^^,^^10^

Nucleosome Remodeling and Deacetylase (NuRD) and SWItch/Sucrose Non-Fermentable (SWI/SNF) complexes are 2 prominent chromatin remodelers that have been extensively studied in the context of homeostasis and cancer.^11-15^ They exhibit distinct, yet interconnected functions and compete for binding to specific genomic loci, thereby influencing gene expression patterns. The primary function of the NuRD complex is to remove acetyl groups from histones, which leads to chromatin compaction and transcriptional repression. Instead, the SWI/SNF complex disrupts histone-DNA contacts and mobilizes nucleosomes, thereby promoting the accessibility of DNA to transcription factors and promoting gene expression.^14^^,^^16^^,^^17^

In a previous study from our laboratories, the expression and functional analysis of the *CDK2AP1* gene (also known as *DOC1*, Deleted in Oral Cancer), encoding for a key NuRD subunit, has revealed its central role in the “*tug-of-war*” between the NuRD and SWI/SNF chromatin remodeling complexes for the activation/repression of master regulators of epithelial-to-mesenchymal transition (EMT).^18^ Notably, the same competition was previously shown to play a regulatory role in contexts other than cancer, namely, B cell development,^19^ vascular Wnt signaling,^20^ and the inflammatory response.^21^

Here, we show that the same NuRD-SWI/SNF competition controls NF-kB activation together with other well-known inflammatory pathways, which underlie the coordinated regulation of EMT and inflammation in OSCCs.

In the heterotypic model of cancer, malignant cells are embodied within their tumor microenvironment (TME), which encompasses a broad spectrum of diverse cell types, including stromal, endothelial, and immune cells, all of which play an active role in supporting and driving tumor progression.^6^ Immune cells such as monocytes are recruited into the surrounding extracellular matrix (ECM) through cytokines and chemokines secreted by cancer cells. Interleukin 6 (IL-6) and chemokines such as CCL2, CCL3, CCL4, and CCL5 contribute to monocyte recruitment and polarization towards tumor-associated macrophages (TAMs).^22-24^ TAMs exhibit remarkable plasticity and can adopt distinct activation states, including the classically activated pro-inflammatory M1 phenotype and alternatively activated anti-inflammatory M2 phenotype. The balance between M1 and M2 macrophages within the TME is critical for tumor development, invasion, and immune evasion.^25^

Here, we show how, in OSCC, the competition between NuRD and SWI/SNF chromatin remodeling complexes, next to the coordinated activation of EMT and inflammation, affects TME composition and, in particular, macrophage recruitment and polarization, thus further modulating tumor behavior. The elucidation of the underlying regulatory networks and signaling pathways is likely to reveal novel therapeutic strategies centered on the NuRD-SWI/SNF axis and meant to target, in coordinated fashion, EMT in cancer cells and suppress inflammation and macrophage polarization in the TME.

## Methods

### Cell lines

The OSCC cell lines CA1 and LM, provided by A.B., were cultured as previously described.^26^^,^^27^ The HEK293T cell line obtained from ATCC was cultured in DMEM (Thermo Fisher Scientific, #11965092) supplemented with 10% FBS, 1% penicillin/streptomycin (Thermo Fisher Scientific, #15140122), and 1% glutamine (Gibco #25030024). All cells were maintained in a humidified atmosphere at 37°C with 5% CO_2_.

### Generation of CDK2AP1 knock-out clones

Single guide RNAs targeting CDK2AP1 were designed using the CHOPCHOP web tool (http://chopchop.cbu.uib.no/). The top 3 outputs, sgRNA1 (5′-TTCACGCTAGAGGACTGGTT-3′), sgRNA2 (5′-AAGCAAATACGCGGAGCTGC-3′), and sgRNA3 (5′-GGTGCCCCAAAGCAAATACG-3′), were cloned into the TLCV2 lentiviral vector (Addgene #87360). Lentiviruses were produced by co-transfection of HEK293FT cells (Invitrogen) with the lentiviral expression vector TLCV2 and psPAX2 (Addgene #12260) and pMD2.G (Addgene #12259) plasmids encompassing the envelope glycoprotein VSV-G (vesicular stomatitis virus G protein). Viral supernatants were collected at 48-72 h after transfection. OSCC cell lines were then infected with lentiviruses, followed by selection using 1 µg/mL puromycin (Invivogen) for 5 days. Cas9-GFP was induced for 5 days with 1 µg/mL doxycycline. Next, GFP-positive cells were single-cell seeded in 96-well plates and the resulting clones were screened for CRISPR/cas9 traces in the proximity of the sgRNA by DNA sequence analysis (primers: FW: 5′-TTTGCTGAACCCATTTCTTTCT-3′; REV: 5′- ATTTTCCCCAAAAGTCTTTCCA-3′) as well as by protein detection by immunoblot analysis. Of the 3 sgRNAs, sgRNA3 was the most efficient for the generation of CDK2AP1 KO clones.

### Generation of inducible shRNA-expressing cells

To knockdown *CDK2AP1* expression, lentiviral-inducible shRNA vectors were purchased from Horizon Discovery Ltd (Clone Id: V3THS_410413). For lentivirus production, the shRNA constructs were packaged into second-generation virus particles using psPAX2 (Addgene #12260) and pMD2.G (Addgene #12259) in HEK293T cells. Viral supernatants were collected at 48-72 h after transfection. OSCC cell lines were then infected with lentiviruses in the supernatants, and the pools were selected using 1 µg/mL puromycin (Invivogen) for 5 days. shRNA expression was induced using 1 μg/mL doxycycline at different time points. The extent of induction was assessed by flow cytometry, whereas *CDK2AP1* downregulation was assessed by RT-qPCR and immunoblotting.

The same approach was employed to generate inducible shRNAs against *BRM* (Clone ID: V3THS_372090) and *BRG1* (Clone ID: V3THS_317182; horizon discovery).

### RT-qPCR and PCR analyses

Total RNA was isolated using TRIzol reagent (Thermo Fisher Scientific #15596018) and reverse-transcribed using a high-capacity cDNA reverse transcription kit (Life Technologies #4368814), according to the manufacturer’s instructions. RT-qPCR was performed using the Fast SYBR Green Master Mix (Thermo Fisher Scientific) on an Applied Biosystems StepOne Plus Real-Time Thermal Cycling Research system with 3 replicates per group. The relative gene expression was determined by normalizing the expression of each target gene to that of GAPDH. The results were analyzed using the 2-(ΔΔCt) method. The specific RT-qPCR primers used are listed in the Supplementary Materials and Methods.

### Immunoblot analysis

Cells were lysed in 10 mM Tris buffer pH 7.5 containing 1% SDS, supplemented with complete Mini Protease Inhibitor Cocktail (Roche #11836153001), and subjected to sodium dodecyl sulfate (SDS)- polyacrylamide gel electrophoresis (PAGE). Phosphatase inhibitor cocktails (Roche #04906837007) were added to the lysis buffer to detect phosphorylated proteins. Protein quantification was performed using a BCA Protein Assay Kit (Millipore), and 10 µg of protein was loaded for each sample. The membranes were incubated with primary antibodies against CDK2AP1 (1:500, Santa Cruz Biotechnology #sc-390283), β-actin (1:2000, Cell Signaling #8457), p65 (rabbit, 1:500; Abcam #ab16502), P^s536^p65 (rabbit, 1:250; Abcam #ab86299), BRM (1:1000, Cell Signaling #11966), and BRG1 (1:1000, Cell Signaling #49360), followed by incubation with polyclonal goat anti-mouse/rabbit immunoglobulin horseradish peroxidase (HRP)-conjugated secondary antibodies (Dako) diluted 1:1000 for 1 h at room temperature. The signals were detected with the Pierce ECT immunoblotting substrate (Thermo Fisher Scientific) using Amersham 5 AI600 (GE Healthcare, USA).

### Immunofluorescence microscopy and data analysis

Coverslips coated with a monolayer of cultured cancer cells were fixed for 15 min in 4% PFA at room temperature and washed twice with PBS. Cells were first permeabilized for 15 min at room temperature with 0.1% Triton X-100 and then incubated in blocking buffer (5% milk powder in PBS-Tween) for 1 h at room temperature. The cells were then incubated overnight at 4°C with primary antibodies against CDK2AP1 (rabbit, 1:500; GR788 In-house production),^18^ p65 (rabbit, 1:800; Cell Signaling #6956), and P^s536^p65 (rabbit, 1:800; Cell Signaling #3033). After washing twice with PBS-Tween, coverslips were incubated for 1 h at room temperature in a blocking buffer containing goat anti-rabbit/mouse Alexa Fluor 568/488 conjugate (1:250; Life Technologies #A-11011) secondary antibodies. Cells were counterstained with DAPI to visualize the nuclei and Phalloidin Alexa Fluor 647 (1:200, #A-12379, Life Technologies) to visualize actin filaments. The coverslips were mounted in VECTAHIELD HardSet Antifade Mounting Medium (Vector Labs #H-1400) and imaged using a Zeiss LSM-700 confocal microscope. Images were processed using ImageJ software (National Institutes of Health, Bethesda, MD, USA).

For quantification purposes, the DAPI channel was used to identify cell nuclei and converted to binary following Gaussian blur in ImageJ. Mean fluorescent intensity was measured with the analyze particle function. Data were imported in R and cells were filtered based on their nuclear area (<400 a.u.) and circularity (> 0.8). The remaining cells were visualized across experimental groups and *P* values were added with respective to the parental control using the Wilcoxon test.

### Immunohistochemistry

Tissues obtained from animal experiments were fixed overnight in 4% PFA and embedded in paraffin. Paraffin blocks containing human cancer tissue were obtained from the Department of Pathology at the Erasmus Medical Center in Rotterdam. Four-micrometer sections were mounted on slides. IHC was performed using the EnVision Plus-HRP system (Dako) and antibodies directed against anti-human mitochondria (1:100, Merck #1273), CDK2AP1 (rabbit, 1:500; GR788 In-house production),^18^ ECAD (1:1000, Cell Signaling #24E10;), NCAD (1:1000, Cell Signaling #D4R1H), and FN1 (1:1000, Cell Signaling #E5H6X). Briefly, paraffin-embedded sections were dewaxed with xylene and hydrated in 100% and 70% ethanol. Antigen retrieval was performed using pressure cooker pretreatment in Tris-EDTA buffer (pH 9.0). Subsequently, slides were incubated at room temperature with 3% hydrogen peroxidase for 10 min to block endogenous peroxidase activity. Tissue sections were washed and blocked with 5% milk in PBS-Tween for 1 h and incubated with primary antibodies overnight at 4°C. The slides were washed twice with PBS-Tween and incubated with rabbit EnVision+ System HRP (K4001, Dako) or mouse EnVision+ System HRP (K4007, Dako) for 1 h. Tissue slides were counterstained with Mayer’s hematoxylin. Dehydration was performed by incubation in 70% and 100% ethanol followed by xylene before slides were mounted using Pertex (Histolab #00811).

### Collagen migration assays

To monitor the invasive capabilities of OSCC cells, parental cell lines and their CDK2AP1 KO counterparts were plated in 2 different age models.

In the collagen droplet model, invasive behavior and morphological changes were evaluated. Briefly, 5 × 10^4^ cells were seeded in a 20 μL droplet of rat tail collagen type 1 (4 μg/mL). Images were taken 4 days after plating.

In the collagen chamber model, the invasive capacity was assessed using the maximum penetration measured after 5 days. Cells were seeded on top of a rat tail collagen type 1 gel (4 μg/mL) in a scaffold at a density of 20 × 10^4^ cells/well in 24-well plates. After 5 days of incubation, gels were harvested, fixed in PFA 4%, embedded in soft agar, processed, and sectioned for immunohistochemistry. Max distances were calculated using QuPath version 0.4.0.

### Animal experiments

All protocols involving animals were approved by the Dutch Animal Experimental Committee and conformed to the Code of Practice for Animal Experiments in Cancer Research established by the Netherlands Institute for Health Protection, Commodities, and Veterinary Public Health (The Hague, the Netherlands, 1999). Animals were bred and maintained in the Erasmus MC animal facility (EDC) under specific pathogen-free (SPF) conditions.

Subcutaneous injections were administered to 6- to 8-week-old NSG (NOD.Cg-Prkdcscid Il2rgtm1Wjl/SzJ) male and female mice. Briefly, 5 × 10^5^ cells were re-suspended in 50 μL of culture medium and injected subcutaneously (4 injections in the flanks of each animal). Tumor growth was monitored by one of the authors (R.S.) and animal caretakers. The mice were sacrificed when the tumor size reached the humane endpoint. Upon collection, primary tumors were fixed, processed, and scanned with the Zeiss Axioscanner 7.0 using 20× magnification.

### Chemokine and cytokine assays

Parental and CDK2AP1-KO cell lines were seeded at a density of 1 × 10^5^ cells in 10 cm dishes. Supernatants were collected after 96 h, and aliquots were stored at −80°C. Chemo- and cytokine concentrations were determined using the Legendplex bead assay (BioLegend). For this project, the LEGENDplex Human Macrophage/Microglia Panel (13-plex) and the LEGENDplex HU Proinflam. Chemokine Panel 1 (13-plex) was used. Bead analysis was performed using a BD FACS Lyric flow cytometer and the data were analyzed using FlowJo V10.6.2.

### PBMC chemotaxis

Peripheral blood mononuclear cells (PBMCs) were isolated from buffy coats obtained from healthy blood donors (Sanquin). Written informed consent for the research use of donated blood was obtained from the Sanquin blood bank. Peripheral blood mononuclear cells were obtained by density centrifugation using a Ficoll Paque PLUS (GE Healthcare). Migration of PBMCs from 3 donors was assessed using 24-well transwell chambers, where the 2 compartments were separated by a 5 μm pore size polycarbonate membrane (Corning, #3421). The upper compartment contained 100 μm of PBMC suspension (1 × 10^5^ cells/well) in RPMI-1640 serum-free medium. The lower compartment contained 500 μL of either cancer cell line derived conditioned medium or control medium (serum free medium; 2% serum control; CCL19 [20 ng/mL, R&D Systems]). PBMCs were allowed to migrate from the upper to the lower chamber for 3 h. At the end of the assay, the upper well was removed and the cells in the lower compartment were analyzed using a cytofluorimeter. Migrating cells were counted to distinguish lymphocytes from monocytes, based on size differences.

### Macrophage maturation and polarization

Monocytes were obtained from PBMC fractions obtained from 6 healthy donors by magnetic-associated cell sorting using CD14^+^ beads, following the manufacturer’s guidelines (Miltenyi Biotec). Sorting purity was confirmed by flow cytometry using a BD Lyric flow cytometer (BD Biosciences).

Monocytes were seeded at a density of 1 × 10^5^ cells per well in 96-well plates and incubated to mature for 6 days in RPMI-1640 medium containing 10% pooled human serum (Sanquin), 1% (v/v) GlutaMAX (Gibco), and 20 ng/mL monocyte colony-stimulating factor (M-CSF, R&D Systems) at 37°C with 5% CO_2_. The medium was replaced on days 2 and 4.

Mature macrophages were stimulated for 48 h with complete medium containing IFN-γ (20 ng/mL, R&D Systems) and LPS (100 ng/mL, Sigma Aldrich), IL-4 (20 ng/mL, R&D Systems), or IL-10 (20 ng/mL R&D Systems), to induce the M1 and M2 phenotypes, respectively. To investigate the effect of CDK2AP1-KO cancer cells on polarization, macrophages were stimulated with their conditioned medium, as well as with the control medium from the CDK2AP1 parental lines. The expression of macrophage phenotypic markers was investigated using flow cytometry. Non-polarized M0 macrophages cultured for 48 h in complete medium without additional cytokines were used as controls for each donor.

Macrophages were dissociated from the wells using Accutase (Merck Millipore) and washed twice with PBS before staining for 30 min with the fixable viability dye ZombieViolet (Biolegend). Cells were fixed with 4% PFA for 15 min, and after Fc receptor blocking with Human TruStain FcX (Biolegend), cells were stained with the following antibodies in FACS buffer (PBS with 2% fetal calf serum, 0.2 mM EDTA, 0.01% sodium azide): anti-CD80-FITC, anti-HLA-DR-APC-Cy7, anti-PD-L1-APC, anti-CD163-PE, anti-CD200R-PE-Cy7, and anti-CD206 BV786 (all from Biolegend). The mean fluorescent intensities were quantified by flow cytometry using a BD Lyric flow cytometer (BD Biosciences), and the data were analyzed using FlowJo V10.6.2. The cell culture supernatant was collected 96 h after seeding 1 × 10^5^ cells in 10 cm dishes and stored at −80°C. Aliquots of the same media were used for quantification of cytokines using the Legendplex Cytometric Bead Assay (Biolegend) as described above.

### Patient cohort, immunohistochemistry, and tumor tissue microarrays (TMA)

Tissues from 100 primary oral squamous cell carcinomas of the tongue surgically removed between 2007 and 2013 were obtained from the tissue bank of the Department of Pathology of the Erasmus Medical Center and recorded within the framework of the RONCDOC project, an initiative undertaken by the RWHHT (Rotterdamse Werkgroep Hoofd-Hals Tumoren). The cohort included patients with tongue squamous cell carcinoma (TSCC) removed by surgery as the primary treatment at the Erasmus MC Cancer Institute. Patients with a history of head and neck cancer were excluded from the study.

Patient characteristics, comorbidities, TNM staging, treatment protocol, histopathological characteristics, recurrent disease, and survival were recorded for all subjects included in this cohort. Tissue preparation and staining were performed as described previously.^28^ Briefly, consecutive 4 μm sections were cut from formalin-fixed paraffin-embedded (FFPE) cancer tissues using a microtome. Hematoxylin and eosin (H&E) and CDK2AP1 immunohistochemical staining (1:250, Santa Cruz #sc-390283) were evaluated and scored by a pathologist. The obtained data were then correlated with the clinicopathological information included in the cohort.

TMAs were obtained from the same FFPE blocks. Three regions of interest for each tissue block (1 in the center of the tumor and 2 in the tumor border) were selected for sampling (1 mm diameter each) and included in the TMA blocks.

### TME analysis by multiplex immunofluorescence

Multiplex immunofluorescence (IF) analysis was performed by automated IF using Ventana Benchmark Discovery ULTRA (Ventana Medical Systems Inc.). Four-micrometer formalin-fixed paraffin-embedded (FFPE) sections were stained. Briefly, following deparaffinization and heat-induced antigen retrieval with CC1 (#950-224, Ventana) for 32 min, anti-CD68 was incubated for 20 min at 37°C, followed by omnimap anti-mouse HRP (Ventana #760-4310) and detection with DCC (Ventana #760-240) for 8 min. Antibody denaturation was performed with CC2 (Ventana #950-123) at 100°C for 20 min. Second, incubation with anti-34BE12 was performed for 4 min at 37°C, followed by omnimap anti-mouse HRP (Ventana #760-4310) and detection with R6G (Ventana #760-244). Antibody denaturation was performed with CC2 (Ventana #950-123) at 100°C for 20 min. Third, anti-CD163 was incubated for 32 min at 37°C, followed by omnimap anti-mouse (Ventana #760-3210) and detection with Red610 (Ventana #760-245). Antibody denaturation was performed with CC2 (Ventana #950-123) at 100°C for 20 min. Finally, the anti-CD14 antibody was incubated for 32 min at 37°C, followed by omnimap anti-rabbit HRP (Ventana #760-4311) and detection with Cy5 (Ventana #760-238) for 8 min. Antibody denaturation was performed with CC2 (Ventana #950-123) at 100°C for 20 min. Finally, the anti-CD3 cells were incubated for 32 min at 37°C, followed by omnimap anti-rabbit HRP (Ventana #760-4311) and detection with FAM (Ventana #760-234) for 8 min. Primary antibody specifications can be found in the Supplementary Materials and Methods. Finally, the slides were washed in phosphate-buffered saline and mounted with Vectashield containing 4’,6-diamidino-2-phenylindole (Vector Laboratories, Peterborough, UK). The slides were imaged using an Axioscan Zeiss microscope.

### TMA bioinformatic data analysis

Three tissue microarrays (TMAs) were constructed with punches from the tumor core and periphery of a cohort of OSCC patients (N = 462 cores, N = 164 tumors). The TMAs were cut to a thickness of 4 µm and stained with antibodies directed against cytokeratin (34BE12), CD3, CD14, CD68, and CD163. Whole slides were imaged with Zeiss Axioscanner 7.0, and analyzed with VIS from Visiopharm (v2023.11). The tumor regions were initially detected using an AI DeepLab algorithm based on the R6G signal. In tumor and non-tumor regions, nuclei and cytoplasm were detected using a second AI U-net algorithm based on the DAPI signal. Next, the individual fluorescent channels were quantified based on the median intensities. Downstream processing was performed in R using SPIAT (v1.0.4). Cells were annotated according to the positivity of the following channels: Cytokeratin+CD- (tumor), CD14+CD3-CD68-CD163- (monocyte), CD68+ (macrophage), CD163+CD68+ (M2 macrophage), and CD3+ (T-cell). Following annotation, cells were visualized based on the center coordinates of their nuclei. Cell type fractions were computed for each core and cohort-wide statistics were performed using the ggpubr package (v0.4.0).

### Bulk RNAseq and bioinformatic data analysis

Cell lines were grown to 60%-70% confluency before RNA extraction was performed using TRIzol. Subsequent DNase treatment was performed using the TURBO DNA-free Kit (Invitrogen) to purify the samples. Samples were then sequenced using the DNA nanoball (DNB) sequencing protocol (BGI) to a depth of 50M reads/sample. The SOAPnuke pipeline (BGI) was used to perform quality checks and preprocessing. Clean reads were mapped to the human reference genome (hg19) using STAR aligner (v2.7.9a) and GENCODE v35 annotation. Duplicates were marked with Sambamba (0.8.0) and gene counting was performed using FeatureCounts (v2.0.0). Downstream analysis was performed in R, using the DESeq2 package (v1.36.0). After variance-stabilizing transformation, principal component analysis was performed using the top 500 variable expressed genes. Differentially expressed genes were visualized using the enhanced volcano package (v.1.14.0). Gene ontology and enrichment analyses were computed based on the hallmark gene sets from the molecular signature database using the enrichR (v.3.1) and fgsea (v.1.22.0) packages, respectively.

### ChIP sequencing data analysis

CHD4 chromatin-binding peaks from ChIP sequencing of the SCC9 cell line overexpressing CDK2AP1 or transfected with an empty vector were retrieved from Modh-Sarip et al work.^18^ BEDOPS^29^ was used to identify consensus peaks for each condition by selecting peaks identified in at least 2 replicates. Peaks were then annotated using the ChIPseeker (version 1.30.3) R package.^30^ Overrepresentation analysis was performed on CHD4 binding peaks present exclusively in the CDK2AP1 overexpressing samples using the clusterProfiler (version 4.2.2) R package^7^ using the MSigDB Hallmarks^31^ annotation.

### Data availability

RNA sequencing data regarding the gene expression profiling of oral squamous cell carcinoma (OSCC) cell lines, either parental or CDK2AP1 knockouts (KO), have been deposited in the Gene Expression Omnibus and are publicly accessible with the identifier GSE260789. Gene expression profiling of the CA1 cell line without silencing BRM and BRG1 can be accessed using the identifier GSE260831.

### Statistical analysis

Results are shown as the mean of 3 biological replicates, with error bars representing the standard error of the mean (S.E.M). The details of each statistical test are provided in the figure legends. All statistical tests and graphs were generated using the R software package.

### Ethics statement

Human tissue and patient data were used according to “The Code of Conduct for Responsible Use” and “The Code of Conduct for Health Research,” as stated by the Federation of Dutch Medical Scientific Societies.^20^ The Erasmus MC Medical Ethics Committee approved the research protocol (MEC-2016-751).

## Results

### CDK2AP1 genetic ablation underlies EMT and increased invasive behavior in OSCC cancer cell lines

**Figure 1.**
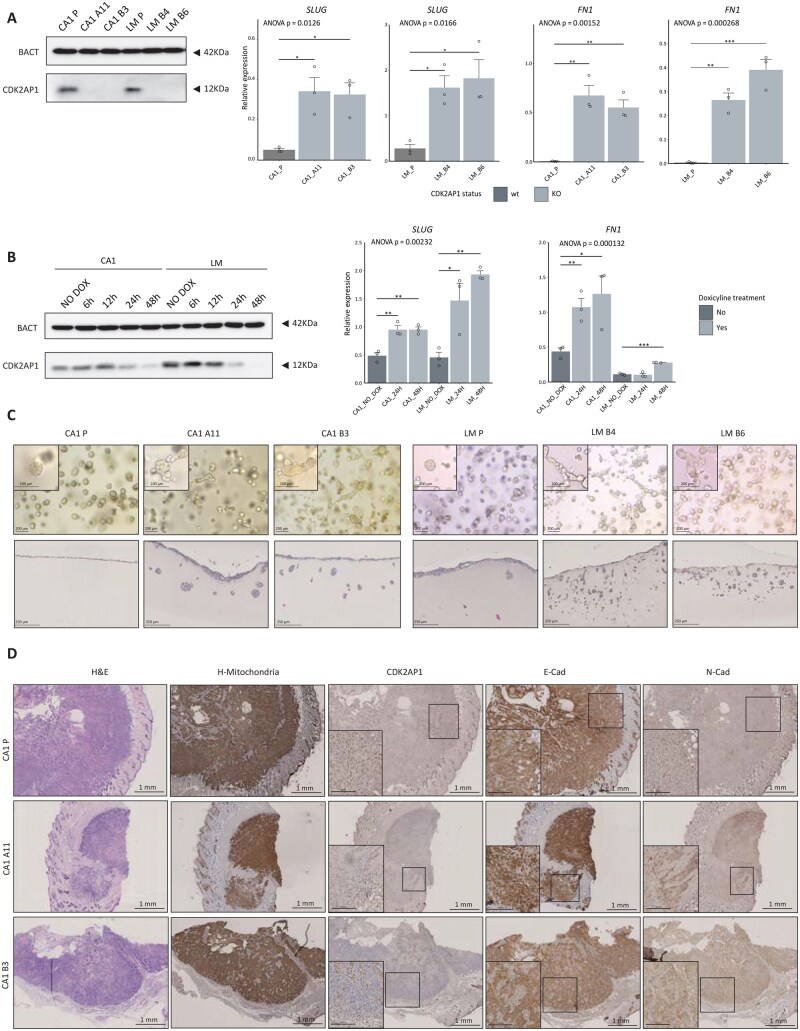
CDK2AP1 ablation in OSCC cell lines underlies epithelial to mesenchymal plasticity. **A**. Left panel: CDK2AP1 immunoblot analysis of our panel of OSCC parental (CA1-P and LM-P) and KO (CA1-A11 and -B3; LM-B4 and -B6) cell lines. The 12 kDa CDK2AP1 protein was observed exclusively in the parental cell lines. β-Actin (BACT) was used as the loading control. The blots shown here are representative examples of 3 independent experiments. Right panel: RT-qPCR expression analysis of the EMT transcription factor *SLUG* and the EMT marker fibronectin (*FN1*) in CDK2AP1-proficient and -deficient cell lines. Experiments were performed in triplicate, and mRNA expression was normalized to that of GAPDH. *P* values denote 1-way ANOVA and 1-sample t-tests against the parental cell lines (*P<.05, **P<.01, ***P<.001). **B**. Left panel: CDK2AP1 immunoblot analysis of CA1 and LM OSCC cell lines transduced with *CDK2AP1*-shRNA and induced with doxycycline (DOX) at different time points. β-Actin (BACT) was used as the loading control. The blots shown here are representative examples of 3 independent experiments. Right panel: RT-qPCR analysis of the above EMT markers in *CDK2AP1*-shRNA cells at the same time points evaluated in the immunoblot depicted in **A**. Experiments were performed in triplicate, and mRNA expression was normalized to that of GAPDH. *P* values denote 1-way ANOVA and 1-sample t-tests against the NO-DOX condition (*P<.05, **P<.01, ***P<.001). **C**. Analysis of parental and *CDK2AP1*-KO OSCC cell lines grown in rat-tail type I collagen medium. The top panels depict images of cells resuspended in single cells and grown in 3D collagen matrix. Images were taken after 72 h in culture to evaluate morphological differences. Higher-magnification images (20×) are shown in the top-left corner inlet. Scale bars correspond to 200 μm. The bottom panels show histological sections stained with hematoxylin and eosin (H&E) relative to the invasion assay. The cells were seeded on top of the collagen layer and allowed to grow through the matrix. The images correspond to the 5th day of culture. Scale bars correspond to 250 μm. **D**. Immunohistochemistry (IHC) analysis of tumors obtained by subcutaneous transplantation of either parental or KO CA1 cells in NSG-recipient mice. Apart from H&E (first column), the sections were stained with antibodies directed against human mitochondria, CDK2AP1, E-cadherin (E-Cad), and N-cadherin (N-Cad). Higher magnification images (20×) of specific tumor areas are shown in the inlets in the bottom-left corners. Lower magnification (4×) scale bars: 1 mm; higher magnification scale bars: 250 μm.

Previously, we reported the key role played by the *CDK2AP1* (*DOC1*) gene in the NuRD-dependent regulation of EMT in competition with the SWI/SNF complex in the context of oral squamous cell carcinoma (OSCC).^18^ Here, in order to develop new cell models to further extend the functional analysis of the consequences of genetic ablation, we employed the *CDK2AP1*-proficient OSCC cell lines CA1 (derived from the floor of the mouth) and LM (mandibular region of the mouth) to generate several knockout (KO) clones by CRISPR/Cas9 technology (Figure 1, A, left panel). RT-qPCR analysis of the EMT-related genes *SLUG* (*SNAI2*) and fibronectin (*FN1*), which were previously shown to have enhanced NuRD gene promoter binding affinity,^18^ revealed their significant upregulation upon *CDK2AP1* ablation in both cell lines (Figure 1, A, right panels). Additionally, flow cytometry analysis revealed reduced E-cadherin (ECAD) expression accompanied by the appearance of N-cadherin (NCAD) in *CDK2AP1*-KO clones when compared with the parental cell line (Figure S1, A).

To further confirm the specific role of *CDK2AP1* in the regulation of *SLUG* and *FN1* expression, we performed knockdown experiments in the parental CA1 and LM cell lines using an inducible short hairpin RNA (shRNA) (Figure 1, B, left panel, and Figure S1, B). Time-dependent RT-qPCR analysis clearly showed progressive and direct upregulation of the *SLUG* and *FN1* target genes upon *CDK2AP1* knockdown (Figure 1, B, right panel). These results validate the role played by *CDK2AP1* in controlling the expression of EMT-associated genes and the overall functional impact of its loss. Next, we assessed the motility and invasive capabilities of the *CDK2AP1*-KO clones. Single cells were embedded in 3D rat-tail type I collagen drops, and their morphologies were monitored after 72 h. While the parental cell lines formed smooth and rounded spheroids, KO clones from both cell lines presented as collective elongated strands with numerous protrusions, indicating increased motility, suggestive of pronounced invasive behavior (Figure 1, C, upper panels). Subsequently, we conducted an invasion assay by seeding cells on top of the collagen layer. Invasion events were scored after 5 days of culture (Figure 1, C, lower panels). H&E staining of the fixed collagen scaffolds revealed that the parental CA1 cell line grew predominantly as a monolayer on collagen, with minimal penetration and a maximum average depth of 91 μm. In contrast, the A11 and B3 *CDK2AP1*-KO clones showed the ability to penetrate the collagen, forming islets of invading cells with average maximum depths of 283.05 and 297.02 μm, respectively (Figure S1, C). The LM clones exhibited even more extreme phenotypes, with extensive clusters of disseminating tumor cells detected inside the collagen, reaching a depth of 498.98 and 311.43 μm for LM-B4 and LM-B6, respectively. The invasive events of the LM parental cell line were fewer, penetrating an average depth of 137.84 μm inside the matrix (Figure S1, C).

To evaluate the consequences of *CDK2AP1* loss in vivo, we subcutaneously injected CA1 parental cells and the corresponding *CDK2AP1*-KO clones into immunocompetent recipient NSG (NOD scid gamma) mice. The parental CA1-derived lesions exhibited increased growth rates when compared to their KO clones (31 days until the animal reached the humane endpoint vs 48 and 51 days for A11 and B3), respectively. Increased tumor-forming efficiency was also observed in the parental CA1 line compared to the *CDK2AP1*-depleted clones (8/8 vs 4/8 upon injection of 5 × 10^5^ cells). As shown in Figure 1, D, immunohistochemistry (IHC) analysis of the resulting tumors showed abundant and strictly membranous expression of ECAD in CA1 parental-derived tumors, both at the edges and in the center of the lesions. In contrast, ECAD was predominantly internalized in the cytoplasm of tumors derived from *CDK2AP1*-KO clones, particularly in cells located along invasive fronts. Furthermore, *CDK2AP1*-deficient tumors exhibited areas positive for NCAD expression, indicating a more mesenchymal-like phenotype. This observation was supported by enhanced fibronectin (FN1) deposition by cancer cells in the *CDK2AP1* KO tumors compared to the parental-derived tumors, where the majority of FN1 staining originated from mouse cells, as indicated by the lack of human mitochondrial staining (Figure S1, D).

Overall, these initial findings support EMT induction upon CDK*2AP1* ablation in OSCC.

### CDK2AP1 deletion promotes activation of pro-inflammatory pathways

To assess whether the genetic depletion of *CDK2AP1* and the consequent alteration of NuRD-SWI/SNF competition results in downstream epigenetic modifications at genomic loci other than those involved in EMT, we conducted an extensive analysis of the ChIPseq data from the Modh-Sarip study.^18^

**Figure 2.**
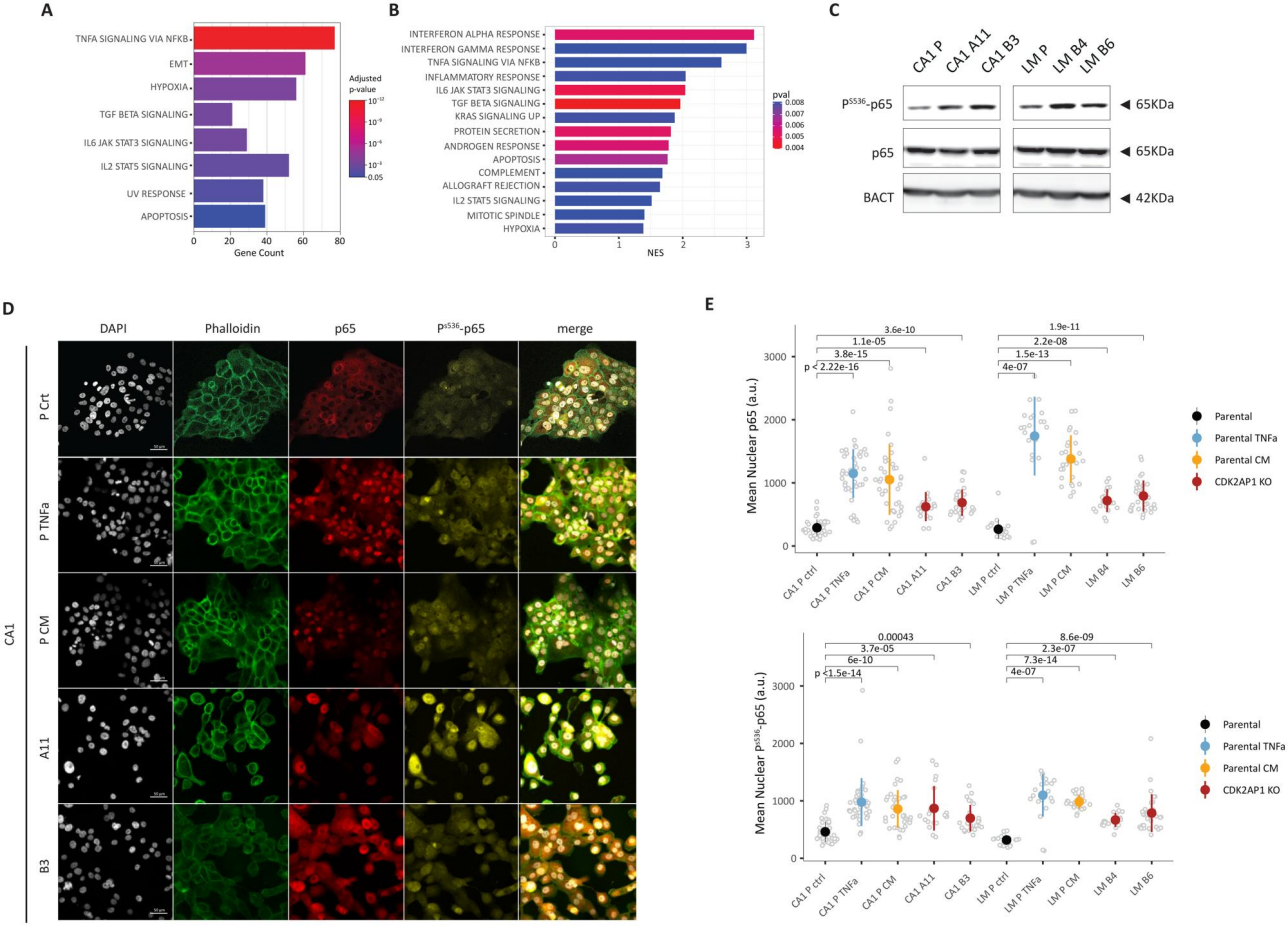
CDK2AP1 deletion promotes inflammation via phosphorylation of p65. **A**. Overrepresentation analysis (ORA) of CHIP-seq data relative to CDK2AP1-dependent NuRD peaks obtained from Modh-Sarip et al.^18^ The analysis was centered on gene promoter regions (<1 kb from the transcription start site [TSS]). Only significantly altered pathways are depicted (*P* < .05). **B**. Gene set enrichment analysis (GSEA) relative to expression profiles obtained from parental and KO CA1 and LM cells. Significantly altered pathways (NES >1 and *P* < .01) are shown. **C**. p65 immunoblot analysis in parental and *CDK2AP1*-KO CA1 cell lines. Both pan- and P-specific antibodies were employed to detect the abundance of the S536 (P^S536^-P65) phosphorylated fraction. Experiments were performed in triplicate, and β-actin (BACT) was used as a loading control. **D**. Representative immunofluorescence images of parental and *CDK2AP1*-KO CA1 cell cultures under normal conditions (Ctr/A11/B3) after treatment with 10 ng/mL TNF-α (TNFa) or with condition medium (CM) from the *CDK2AP1*-KO clones for 1 h. Cells were fixed with 4% paraformaldehyde and stained with antibodies against p65 (red), or phosphorylated P^S536^-P65 (yellow). Nuclei and actin filaments were visualized using DAPI staining and phalloidin, respectively. Scale bar: 50 µm. **E**. Dot plots showing quantification of the immunofluorescence data from panel D and Figure S2, E. The upper graph depicts nuclear p65 abundance, while the lower graph shows P^S536^-p65 levels in CDK2AP1-KO and parental cell lines. *P* values were calculated using the Wilcoxon test.

Overrepresentation analysis (ORA) of CDK2AP1-dependent NuRD chromatin binding peaks revealed an enrichment of inflammation-related pathways, particularly TNF-α/NF-κB (*P* adj < .01) and IL-6-JAK-STAT3 signaling (*P* adj < .01; Figure 2, A), at levels comparable to those of EMT-related pathways. To validate this observation, we performed RNA-seq analysis and compared the transcriptional profiles of the parental cell lines CA1 and LM with their respective *CDK2AP1*-KO clones. Although the limited number of parental (control) samples does not allow any cell line-specific statistically validated conclusion to be drawn, principal component analysis (PCA) of the RNAseq data provided indication of the significant differences between parental lines and, more significantly, on the newly generated *CDK2AP1*-KO clones: while the major variance component (43%) was attributed to the cell line identity (CA1 vs LM), the impact of CDK2AP1 status was evident in the second dimension (28%; Figure S2, A). Differential expression analysis between the 2 groups (parental vs KO; Figure S2, B) confirmed that *CDK2AP1* ablation triggered dysregulation at the gene expression level of multiple inflammation-related pathways, including interferon responses (*P* adj < .005), TNF-α signaling via NF-κB (*P* adj < .01), and IL6-JAK-STAT3 signaling (*P* adj < .005), consistent with the results of ChIP-seq data analysis (Figure 2, B). These findings, notwithstanding the limited number of control samples from the parental cell lines, emphasize the crucial role of CDK2AP1 in regulating the expression of downstream NuRD target genes associated with inflammatory pathways and provide additional insights into the functional consequences of its loss.

In order to identify the transcription factors (TFs) responsible for the upregulation of these pathways, we performed motif analysis of the promoter regions of upregulated genes upon *CDK2AP1* ablation using HOMER.^32^ This analysis identified several interferon regulatory factors (IRFs) and p65 as major TFs implicated in the activation of downstream target genes (Figure S2, C). To validate these findings, we assessed the levels and subcellular localization of the active form of p65, marked by serine 536 (PS536-p65) phosphorylation. Immunoblot analysis confirmed increased levels of PS536-p65 in CA1 and LM *CDK2AP1*-KO clones, while the overall p65 protein expression levels remained unchanged (Figure 2, C).

Immunofluorescence staining further supported these findings: a statistically significant increase in the nuclear abundance of both p65 and its active P^S536^-p65 form was observed in CDK2AP1 KO clones of both cell lines under normal culture conditions (Figure 2, D and E and Figure S2, D and E). More specifically, the nuclear translocation of p65 and P^S536^-p65 observed in the KO clones when compared to the parental cells is indicative of increased activation of NF-κB signaling in the absence of CDK2AP1. Of note, conditioned medium (CM) from the CDK2AP1 KO clones, when employed to culture the parental cell lines, mimics the effect of TNF-α stimulation, resulting in comparable levels of p65 nuclear localization and activation. These findings suggest that the CDK2AP1-KO clones promote autocrine and paracrine secretion of TNF-α, which drives the observed inflammatory responses.

Collectively, our findings provide compelling evidence for the direct involvement of *CDK2AP1* in the regulation of inflammation-related pathways in OSCC, as shown by the expression pattern of key NuRD downstream target genes, likely mediated by p65 nuclear translocation and activation, as well as by TNF-α and interferon signaling stimulation. These results highlight the multifaceted role of CDK2AP1 in both the regulation of EMT and in shaping the inflammatory microenvironment, underscoring its significance in OSCC progression.

### The secretome of CDK2AP1 KO cells promotes recruitment of PBMCs and polarization of macrophages towards a M2-like state

**Figure 3.**
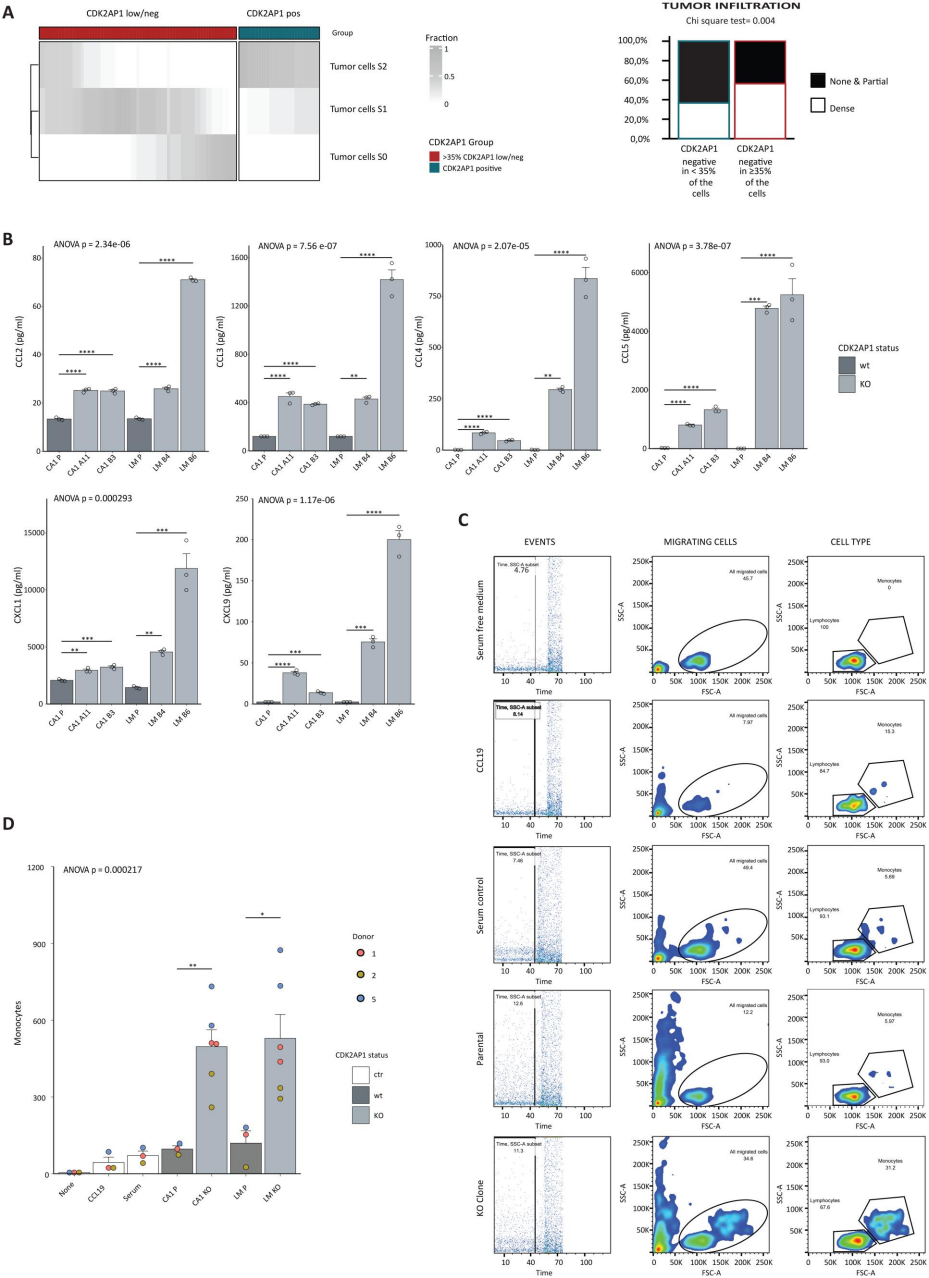
CDK2AP1 ablation results in increased immune-infiltration in vivo and PBMC chemotaxis in vitro. **A**. Left panel: distribution of patient-derived OSCCs based on CDK2AP1 IHC staining intensity. A pre-established 35% threshold was employed to resolve low/negative from positive tumor cells (see also Figure 3, A). For each patient, the fraction of cancer cells for each staining intensity for CDK2AP1 (S0, S1, and S2) is reported in gray scale. The tumors (n=100) were obtained from the RONCDOC patient cohort (for additional details, see Stabile et al.^33^ and the Methods section of the present study). Right panel: Stacked bar plot showing the proportion of tumor infiltration (*none or partial* vs. *dense infiltration*) across the CDK2AP1 positive/low-negative patient groups. **B**. Quantification of chemotaxis-relevant cyto- and chemokines by cytometric bead assay in cell culture medium (for more details, see Methods). Experiments were performed in triplicate; *P* values denote 1-way ANOVA and Tukey’s test against the parental cell line (*P<.05; **P<.01; ***P<.001). **C**. *In vitro* analysis of PBMC chemotaxis. The migration of PBMCs (obtained from 3 independent healthy donors) was assessed using a transwell assay (for more details, see Methods). PBMCs were allowed to migrate from the upper to the lower chamber for 3 h. The lower chamber contained one of the following media: serum-free medium, CCL19-supplemented serum-free medium (20 ng/mL); complete medium including 10% FCS, parental CA1- or LM- conditioned complete media, *CDK2AP1*-KO conditioned complete medium (CA1-A11 and -B3, LM-B4 and -B6). After 3 h, the migrating cells were collected from the lower transwell chamber and quantified by cytofluorimetry. Among the migrating events, lymphocytes were distinguished from monocytes based on their size. Experiments were performed in triplicate with PBMCs from 3 independent healthy donors. **D**. Quantification of PBMC chemotaxis assessed in vitro as described in **C**. *P* values denote 1-way ANOVA and Tukey test of the *CDK2AP1*-KO clones against the respective parental cell line (*P<.05; **P<.01; ***P<.001).

In view of the enhanced inflammatory profile observed upon *CDK2AP1* loss, we examined its relative clinical relevance in OSCC progression and metastasis. To this end, we took advantage of a retrospective cohort of primary oral squamous cell carcinoma of the tongue (n = 100) collected between 2007 and 2013 at the Erasmus MC and including patients who received surgery as the primary form of treatment.^33^ As previously shown,^18^ the majority of tongue tumors show a mixture of CDK2AP1 positive and negative cells, with only a minority of cases demonstrating homogenous loss (Figure 3, A, left panel). Therefore, we established 2 categories of CDK2AP1 immunoreactivity based on the staining intensity, and an optimal threshold of 35% cancer cells negative for CDK2AP1 was found (*P* = .024; see Methods and Figure S3, A). Using this cut-off, tumor immune-infiltration strongly correlated with patients showing more than 35% of tumor cells negative for CDK2AP1 (Chi-square test = 0.004; Figure 3, A, right panel). Based on the latter, we investigated the consequences of CDK2AP1 dysregulation on the tumor microenvironment (TME), particularly on the recruitment of PBMCs and their consequent polarization. It is known that PBMC recruitment plays essential roles in tumor progression and metastasis and that cancer cells are able to recruit them by secreting several chemokines, such as CCL2, CCL3, CCL4, and CCL5 among many others.^34-37^ Accordingly, we established the cytokine/chemokine profile of *CDK2AP1*-KO clones in their culture media (conditioned medium or CM) using antibody arrays (LEGENDplex; see Methods). Detection of the secreted active form of the chemoattractant chemokines confirmed the transcriptomic data with a significantly more pronounced chemoattractant profile in the secretome of the CA1 and LM *CDK2AP1*-KO cell lines than in their *CDK2AP1*-proficient parent clones (Figure 3, B).

CCL2 is one of the most prevalent cytokines expressed in the tumor microenvironment and is considered as one of the major chemoattractants of monocytes/macrophages at sites of inflammation.^22^^,^^38^ Upon *CDK2AP1* deletion, CCL2 secretion increased by at least 2-fold in the KO clones, with a maximum increase of 5.3-fold in the case of the LM-B6 clone when compared with the parental cell lines. Similar chemo/cytokine upregulation was observed for monocyte chemoattractants CCL3, CCL4, CCL5, CXCL1, and CXCL9 (Figure 3, B). Notably, the most extreme differences were observed with CCL5 (RANTES), a central chemokine for monocyte chemoattraction,^39^^,^^40^ when compared with the *CDK2AP1*-proficient cell lines: 36.5- and 60-fold increases in CA1-A11 and -B3, respectively, and striking 1700- and 1870-fold increases in LM-B4 and -B6, respectively (Figure 3, B).

Next, we challenged the ability of the different conditioned media to recruit immune cells using a transwell chemotaxis assay in which PBMCs from 3 independent healthy donors were seeded in the upper chamber while the CM was added to the lower compartment. Serum-free media, unconditioned culture media, and CCL19-supplemented media were used as controls. Migrating cells were collected from the bottom compartments and analyzed by flow cytometry for monocyte quantification (Figure 3, C). CM derived from *CDK2AP1*-KO clones more efficiently attracted monocytes from all 3 donors. In these experimental settings, CA1 and LM KO clones recruited 5.1- (496.33 ± 65.79 vs 96.49 ± 12.73) and 4.4-fold (528.72 ± 93.23 vs 119.60 ± 47.74) more monocytes, respectively, than their parental cell lines (Figure 3, D).

**Figure 4.**
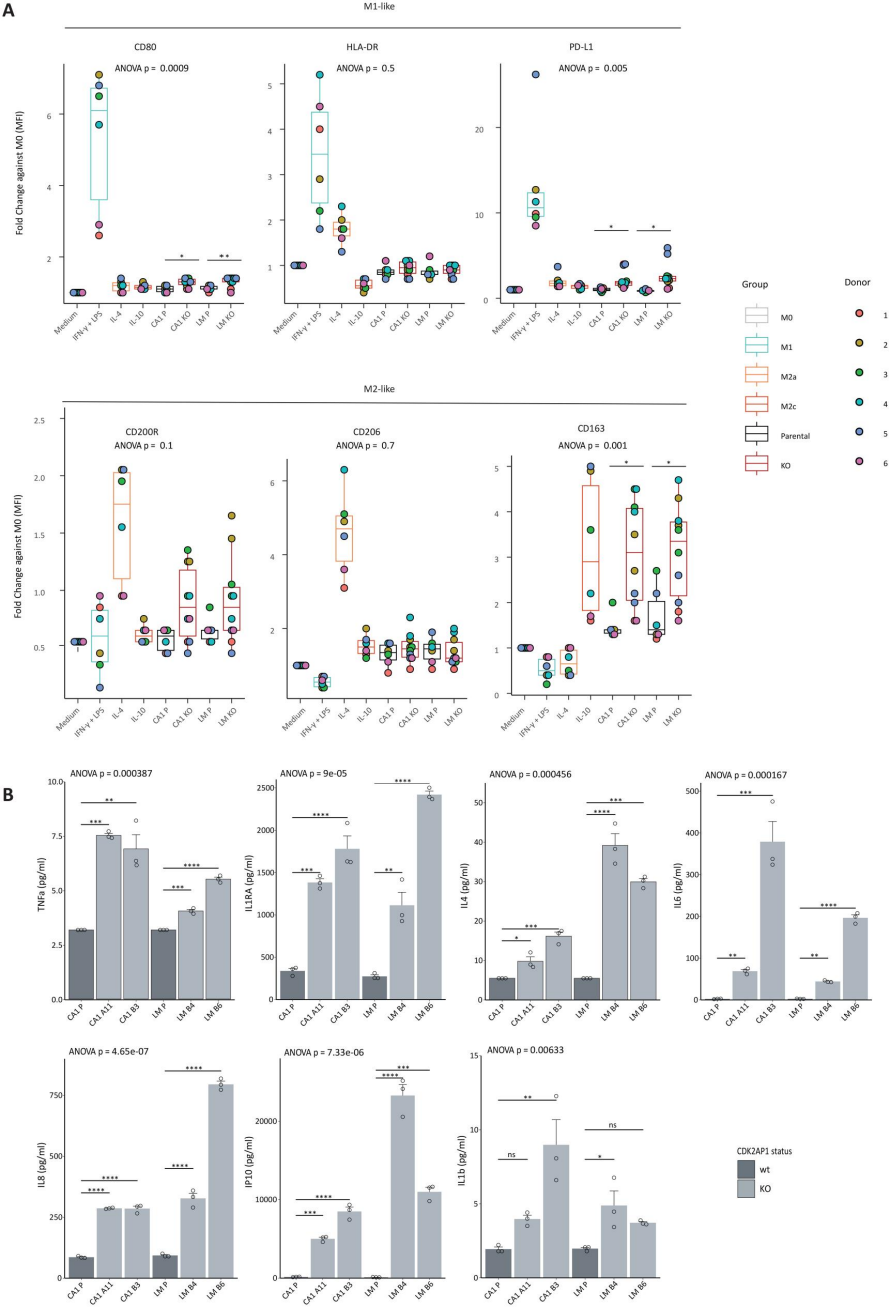
The secretome of CDK2AP1 KO cells promotes polarization of macrophages towards a M2c state. **A**. Expression of M1-like (CD80, HLA-DR, and PD-L1) and M2-like (CD200R, CD206, and CD163) macrophage markers after 48 h polarization with purified cytokines or with the supernatant of the parental and *CDK2AP1*-KO cell lines. PBMCs from 6 independent healthy donors were used (see Methods). *P* values denote 1-way ANOVA and Tukey’s test of the *CDK2AP1*-KO clones against the respective parental cell lines (*P<.05, **P<.01, ***P<.001). **B**. Cyto- and chemokines specific to distinct states of macrophage polarization were quantified using a cytometric bead assay in the supernatant of the culture media of the parental and CDK2AP1-KO cell lines (see Methods). Experiments were performed in triplicate; *P* values denote 1-way ANOVA and Tukey’s test against the parental cell line (*P<.05; **P<.01; ***P<.001).

Subsequently, to investigate the impact of *CDK2AP1* ablation on OSCC cells in inducing macrophage polarization, M0 macrophages from CD14^+^ monocytes from 6 independent healthy donors were stimulated for 48 h with various culture media, including conditioned media from our cell line panel (see Methods). Macrophages were then examined by flow cytometry for the expression of macrophage cell surface differentiation markers. CD80, HLA-DR, and PD-L1 were employed as M1-like markers, while CD200R, CD206, and CD163 were regarded as M2-like markers (Figure S3, B and C). As shown in Figure 4, A, M0 macrophages stimulated to M1 with CM from parental and KO clones only showed statistically significant differences in CD80 and PD-L1 expression, albeit at considerably lower levels than those obtained upon medium complementation with the positive control stimuli IFN-γ and LPS. With regard to M2-like markers, increased levels of CD200R and CD163 were observed upon stimulation with the KO-conditioned media when compared with the parental CM. In the specific case of CD200R, the differences were not significant (ANOVA, *p* = .1), likely due to heterogeneity among the different donors. In addition, the levels of upregulation obtained with the KO CM were significantly lower than those observed with the positive control stimulus IL-4. In contrast, M0 macrophages, upon stimulation with conditioned media from *CDK2AP1*-KO cells, showed a significant increase in CD163 expression levels when compared with the CM from the parental line, ie, similar to that observed upon stimulation with IL-10, a well-established CD163 inducer (Figure 4, A; ANOVA, *P* = .001).

Analysis of cytokine and chemokine secretion from *CDK2AP1*-KO cells, as illustrated in Figure 4, B, revealed a strong statistically significant increase in inflammatory mediator production compared with their *CDK2AP1*-proficient parental counterparts, including TNF-α. This increase aligns with the earlier observation that conditioned medium from *CDK2AP1*-KO cells triggered p65 nuclear translocation and activation in parental cells, mimicking the effect of direct TNF-α stimulation.

Notably, well-established M2 polarization inducers (IL-4, IL-6, and IL-8) were elevated; in particular, IL-6 was previously recognized as one of the most potent drivers of M2-like polarization.^22^^,^^24^^,^^41^

Altogether, these observations strongly suggest that upon *CDK2AP1* loss, OSCC cancer cells secrete inflammatory cytokines and chemokines that efficiently recruit monocytes and polarize macrophages towards the M2-like state, with profound consequences on the tumor microenvironment in support of cancer progression and metastasis formation.

### Convergent pathways, divergent regulations: SWI/SNF loss and CDK2AP1 ablation regulate gene expression in opposite fashion

**Figure 5.**
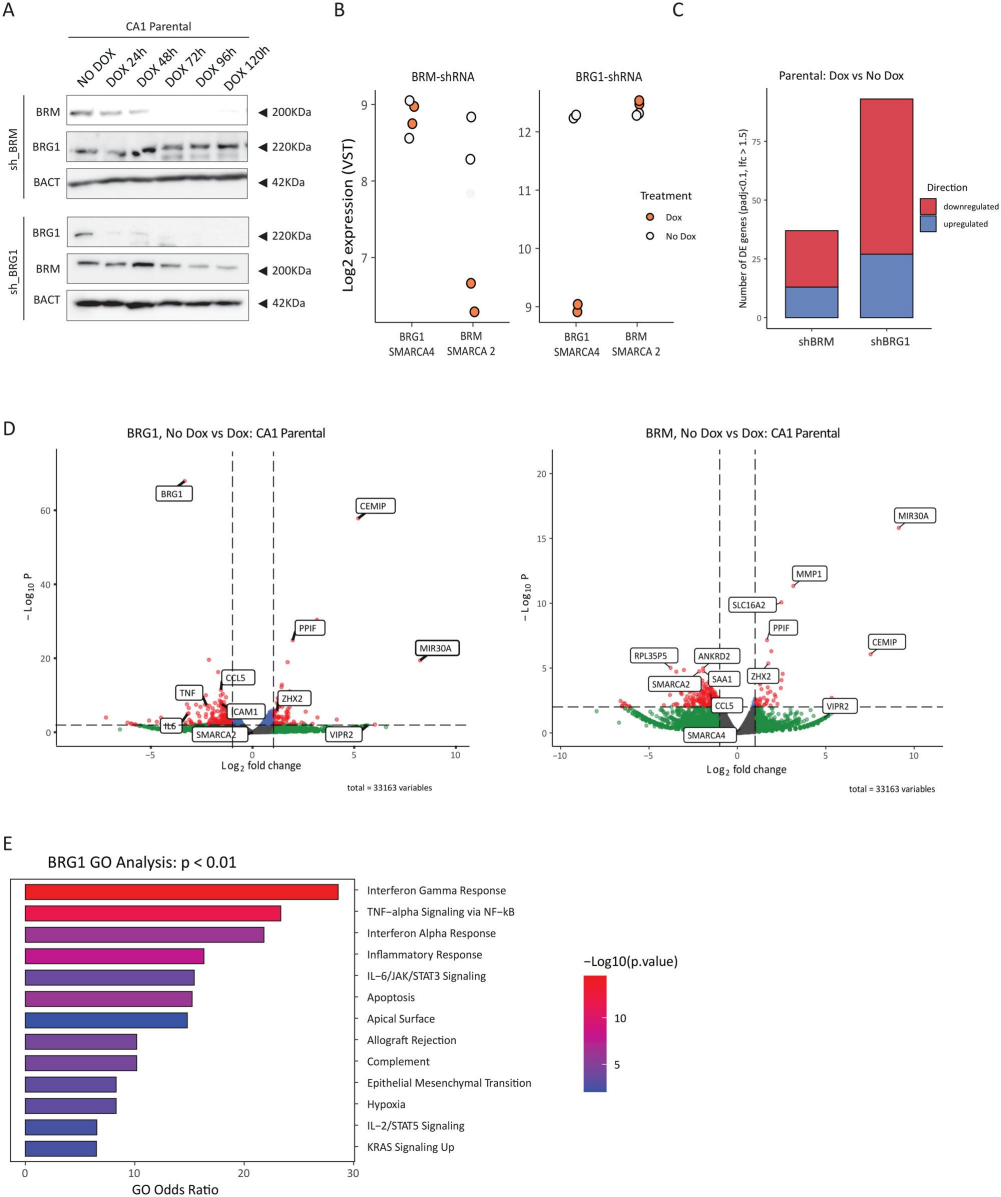
Perturbing SWI/SNF complexes influences the same genes and pathway regulated by CDK2AP1. **A**. BRM and BRG1 immunoblot analysis of CA1 OSCC cells transduced with *BRM*- or *BRG1*-shRNA vectors and induced by doxycycline (DOX) for up to 5 days (120 h). β-Actin (BACT) was used as the loading control. The blots shown here are representative examples of 3 independent experiments. **B**. RNAseq-based validation of the effects of specific *BRM*- and *BRG1*-shRNAs on the expression levels of their respective *SMARCA2* (*BRM*) and *SMARCA4* (*BRG1*) targets. **C**. Overview of differentially expressed genes upon induction with *BRM*- and *BRG1*-shRNAs in the CA1 parental cell line. Only genes with statistical significance (*P* adj. < 0.1 and log fold change>1.5) were up- or downregulated when compared with the uninduced cell line. **D**. Volcano plots relative to differentially expressed genes following *BRG1* and *BRM* knockdown by shRNA. Downward and upregulated genes (log_2_ fold change> 1.5; *P* < .01) are shown on the left and right sides of each plot, respectively. **E**. Gene Ontology Pathway Analysis (GO) of the expression profiles of the CA1 parental cell line upon *BRG1* knockdown (*P* < .01; odds ratio 2).

Competition between NuRD and SWI/SNF complexes has been observed in various cellular contexts, including B-cell lineage specification and vascular Wnt signaling.^19^^,^^42^ Within the scope of our study, the NuRD-SWI/SNF antagonism plays a central role in the coordinated regulation of EMT and the inflammatory response and is critical for tumor progression.^18^^,^^21^ Rather than looking at the chromatin level by means of ChIP analysis of CDK2AP1, a gene notoriously refractory to this approach, we set out to analyze the impact of specific alterations of the SWI/SNF remodeling complex based on the differential expression (Log_2_ Fold change > 1.5) of its downstream target genes. Hence, we assessed whether NuRD and SWI/SNF exert opposing effects on genes involved in inflammatory pathways and the crosstalk between tumor cells and the tumor microenvironment (TME) in OSCC. To this end, we employed inducible short hairpin RNAs (shRNAs) directed against either *BRG1* (*SMARCA4*) or *BRM* (*SMARCA2*), which encode for mutually exclusive ATPase subunits of the SWI/SNF complex, in the CA1 parental cell line. The efficacy of shRNAs in downregulating the protein levels of their target genes was first assessed, as shown in the immunoblot in Figure 5, A. In view of the observed decrease in BRG1 and BRM protein expression in response to doxycycline treatment, and given their partially redundant and cooperative activity,^43-46^ samples were collected for RNA-seq analysis after 24 h of induction with doxycycline (Figure 5, B). As depicted in Figure 5, C, *BRG1* knockdown had a more pronounced impact on downstream gene expression (93 genes with Log2 Fold Change > 1.5, *P* adj < .1; Table S1), when compared with BRM knockdown (37 genes) (Figure 5, C and Table S2). In agreement with its role in transcriptional activation, SWI/SNF knockdown resulted in a majority of downregulated genes (75% for BRG1 and 68% for BRM; Figure 5, C). Analysis of differentially expressed genes (Log_2_ Fold change > 1.5) upon knockdown of the *BRM*-specific SWI/SNF complex (Figure 5, D and Table S2) did not reveal any overlap with the differentially expressed genes previously identified by perturbing *CDK2AP1* expression, with the exception of CCL5. Likewise, the comparative pathway analysis of these datasets did not show any statistically significant result.

In sharp contrast, differential expression analysis of the RNA-seq data obtained upon downregulation of the *BRG1*-specific SWI/SNF complex revealed substantial overlap with the transcriptional changes observed upon *CDK2AP1* genetic ablation, including genes such as *TNF-α*, *IL6*, and *CCL5* (Figures 3, B, 4, B, and 5, D).

Lastly, gene ontology (GO) analysis of the differentially expressed genes upon *BRG1*-KD revealed the enrichment of the same pathways previously identified upon *CDK2AP1* genetic ablation (Figure 2, A and B), including TNF-α/NFκB and IL6-JAK-STAT3 signaling, and EMT (Figure 5, E).

Collectively, these results indicated the central role of BRG1- and CDK2AP1-specific SWI/SNF and NuRD chromatin remodelers in the coordinated deregulation of EMT- and inflammation-related target gene expression in oral cancer cells and inflammation in their microenvironment.

### Tissue microarray multiplex immunofluorescence analysis validates the functional consequences of CDK2AP1 loss on the OSCC TME

**Figure 6.**
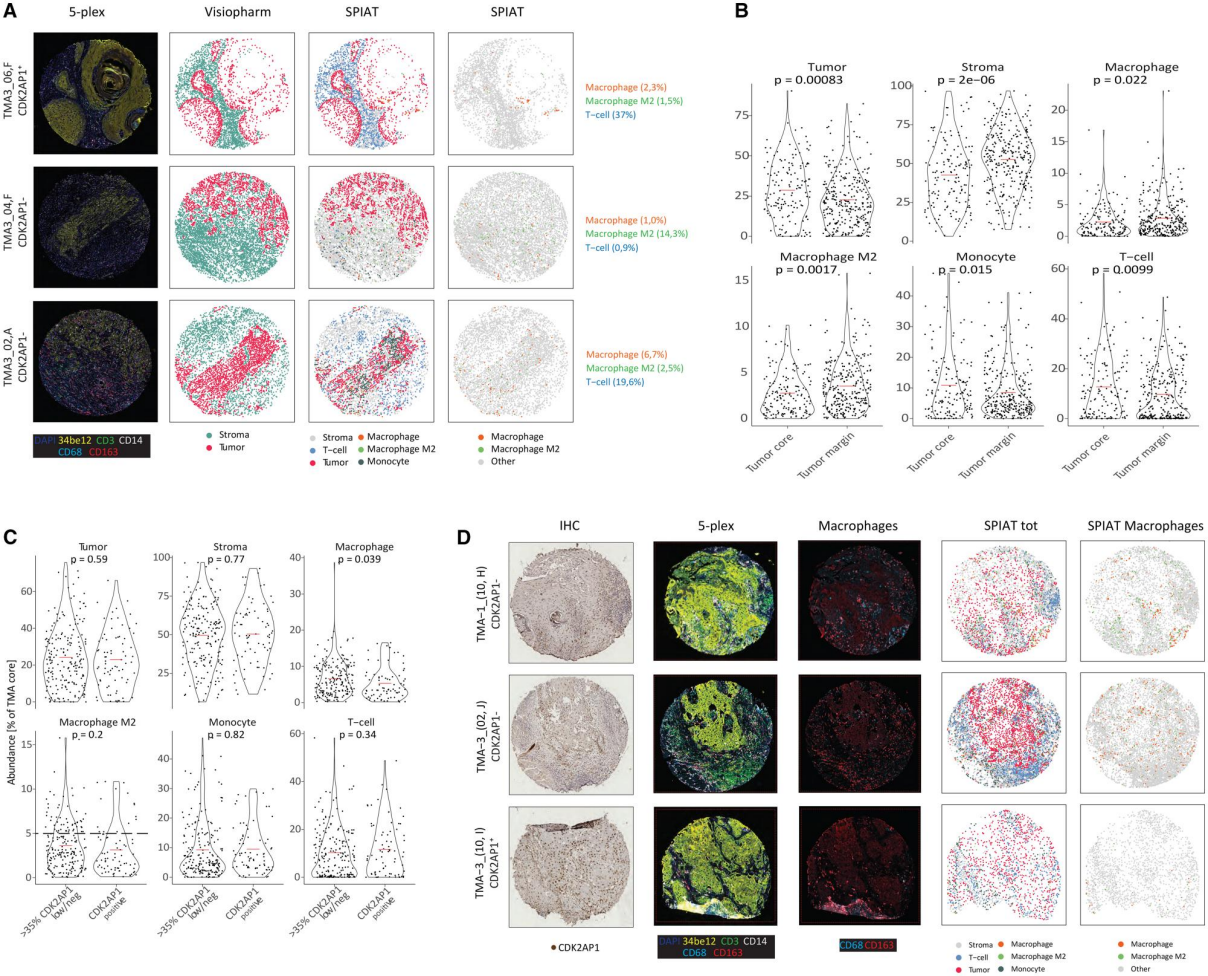
TMA multiplex IF and ISH analysis enable improved characterization and spatial distribution of specific TME markers as a function of CDK2AP1 expression. **A**. Representative images of TMA fields were analyzed for the presence and distribution of different immune cell markers. Next to the IF images (left column), digital reconstructions by Visiopharm and SPIAT of the tumor and stromal cells are depicted. Initial Visiopharm analysis only allowed the resolution of epithelial and stromal (green) cells. Upon SPIAT analysis, T lymphocytes, monocytes, macrophages, and M2c macrophages were resolved. CDK2AP1^-^ refers to CDK2AP1 low/negative cores, whereas CDK2AP1^+^ refers to CDK2AP1 positive cores. **B**. Violin plots relative to the differences in tumor and stromal cells, including (M2c) macrophages, monocytes, and T lymphocytes between the tumor core and tumor margins. *P* values denote paired t-tests. **C**. Violin plot relative to the same parameters analyzed in **B** across CDK2AP1-high and -low/neg cores. The dotted line in the “Macrophage M2-like” graph refers to the 5% of abundance level per TMA core. *P* values denote paired t-tests. **D**. Representative IHC images of CDK2AP1 protein expression in -low/neg (top) and -positive (bottom) TMA tumor cores (left column). Differences in macrophage abundance were shown by IF analysis and SPIAT digital reconstruction. CDK2AP1^-^ refers to CDK2AP1 low/negative cores, whereas CDK2AP1^+^ refers to CDK2AP1 positive cores.

In view of the above results, loss of the *CDK2AP1* tumor suppressor gene in OSCC is expected to have profound consequences on the immune cell landscape of the OSCC TME. To validate our study on patient-derived tumor samples, we conducted multiplex protein immunofluorescence staining on tumor tissue microarrays (TMAs; extensively described in our recent study^33^). Briefly, from a cohort of 141 paraffin-embedded OSCCs, 3 distinct areas of interest were identified in each sample, one in the tumor center and 2 at its periphery. These areas were punched from the blocks to construct TMA slides for multiplex IF analysis. In addition to CDK2AP1, the tissue sections were stained with the following antibodies: CD3 (T-lymphocytes), CD14 (monocytes), CD68 (pan-macrophages), and CD163 (M2-like macrophages).^25^^,^^47^^,^^48^ Furthermore, we utilized an anti-cytokeratin antibody [34BE12], which recognizes cytokeratin 1, 5, 10, and 14, to discriminate between the tumor epithelium (34BE12^+^) and the surrounding stromal tissue (34BE12^-^).^33^ Computational analysis of the digital scans of the stained TMAs, implemented with the recently published SPIAT tool,^49^ allowed us to further improve Visiopharm analysis, not only by resolving the tumor parenchyma from the stroma but also by establishing the composition of immune cells in the TME, their spatial distribution, and relative abundance in relation to CDK2AP1 status. Figure 6, A displays 3 examples of the capabilities of the SPIAT tool to reconstruct the digital versions of IF staining and identify and analyze the different cellular subtypes. In the first row, a core enriched by T-cell infiltration (37% of infiltrating cells) is shown. In the second and third rows, examples of infiltrating CD68^+^/CD163^+^ (14.3%) and high CD68^+^/CD163^—^ macrophages are shown (Figure 6, A).

As shown in Figure 6, B, regardless of CDK2AP1 status, CD68^+^ macrophages were mainly distributed in the periphery (*P* = .02), whereas CD3^+^ lymphocytes were primarily found infiltrating the tumor core (*P* = .009), as were monocytes (CD14^+^; *P* = .015) (Figure 6, B). When considering the CDK2AP1 staining intensity status, the relative proportion of stromal and tumor cells did not significantly change between CDK2AP1-low/negative and positive cores (Figure 6, C). However, CDK2AP1-low/negative tumors exhibited increased CD68^+^ macrophage infiltration (*P* = .039; Figure 6, C). Representative images of the CDK2AP1-positive and -low/negative cases are shown in Figure 6, D.

As for M2-like macrophages (here defined as CD68^+^/CD163^+^), abundance above 5% of the TMA core was only observed in CDK2AP1 low/negative tumors (Figure 6, C, dotted line). However, notwithstanding this trend, these differences were not statistically significant when compared with CDK2AP1-proficient tumors.

In conclusion, our advanced TMA analysis confirmed a direct correlation between CDK2AP1 expression and CD68^+^ macrophage infiltration. The same was not observed for M2-like macrophage polarization identified by CD163 expression, possibly because of technical limitations and/or other potential artifacts.

## Discussion

Although genetic alterations promote tumor initiation, cancer progression is primarily driven by epigenetic and transcriptional reprogramming.^6^^,^^50^^,^^51^ The ability of a cell to alter its identity in response to environmental stimuli, also referred to as phenotypic plasticity, is advantageous over genetic alterations because it enables tumors to rapidly and reversibly respond to the plethora of volatile microenvironmental changes that cancer cells encounter during the multistep metastatic process. Phenotypic plasticity is now widely recognized as the most clinically relevant hallmark of cancer.^6^

Here, we focused on the role of CDK2AP1 in modulating the competition between NuRD and SWI/SNF chromatin remodeling complexes in the coordinated regulation of key downstream signaling pathways that underlie phenotypic plasticity during oral cancer progression and metastasis. Previously, we showed that post-transcriptional mechanisms, including micro-RNA differential expression rather than somatic gene mutations, underlie *CDK2AP1* loss in OSCC.^33^ In addition, an analysis of CDK2AP1 expression in a retrospective cohort of patient-derived OSCCs revealed a strong correlation between CDK2P1 loss and poor prognosis.^33^ Moreover, we showed that deregulation of the competition between NuRD and SWI/SNF leads to the perturbation of EMT/MET dynamics in OSCC.^18^ Here, an extended analysis revealed that *CDK2AP1*, a gene encoding for a subunit of the NuRD complex, is a critical player in OSCC invasion and metastasis, not only because of its role in regulating epithelial-mesenchymal plasticity but also because of its capacity to modulate inflammation and immunosuppression in the tumor microenvironment.

Ideally, ChIP analysis of the CDK2AP1 subunit of the NuRD complex should be the preferred experimental approach to directly investigate chromatin state changes and properly address the aforementioned issues. However, CDK2AP1 has proven refractory to ChIP experiments. This limitation is likely due to steric hindrance or to the inaccessibility of the CDK2AP1 subunit when the NuRD complex is bound to chromatin.

To compensate for this limitation, we instead focused on genetic alterations of the NuRD-specific CDK2AP1 and the SWI/SNF-specific BRG1 (and BRM) subunits and their downstream consequences for EMT and inflammation.

It is now widely accepted that, rather than a complete transition towards a mesenchymal identity, partial or hybrid EMT (pEMT), ie, the co-expression of epithelial and mesenchymal genes, represents a highly metastable state that confers increased invasive and metastatic capacity to cancer cells located at the invasive front.^52^^,^^53^ Notably, scRNA-seq analysis of head and neck squamous cell carcinomas (HNSCC) established pEMT as an independent predictor of locoregional invasion and distant metastasis.^7^ From this perspective, subtle perturbations in the SWI/SNF-NuRD competition, for example, through micro-RNA driven downregulation of the *CDK2AP1* gene as recently shown,^33^ are likely to favor the partial EMT cellular state at the invasive edge of primary oral carcinomas where they directly interact with the TME and trigger a pro-inflammatory state, as shown here.

The coordinated activation of 2 hallmarks of cancer, EMT and inflammation, which are activated through TNF-α/NF-κB, IL-6-JAK2-STAT3, and interferon signaling, underlies phenotypic plasticity and is likely to provide a unique selective advantage to the oral squamous cancer cells during local dissemination and the formation of distant metastases.

Our studies highlight that specific alterations of the NuRD complex, particularly those due to CDK2AP1 loss, simultaneously activate pro-inflammatory signals such as TNF-α while modulating signaling pathways with both pro- and anti-inflammatory consequences (IL-6 and IL-2) to attenuate the immune responses. This apparently contradictory scenario is reflected in the chronically inflamed TME landscape, where cancer cells exhibit dynamic flexibility to exploit both inflammatory and immunosuppressive signals, shaping the most appropriate niche to guarantee tumor growth, immune defense evasion, and the spread of metastases.^35^^,^^54^

Analysis of the cytokine and chemokine secretome activated upon *CDK2AP1* loss revealed complex scenarios where autocrine activation of NF-κB and other inflammation-related pathways is accompanied by paracrine modification of the TME. Of note, Ramirez-Carrozzi et al.^21^ previously reported the competition between the NuRD and SWI/SNF complexes upon inflammation, showing that their antagonism controls macrophage activation upon LPS stimulation through the activation of primary (ie, CCL5 and CCL2) and secondary (ie, IL-6) inflammatory response genes.^21^ By comparing our results with those of the Ramirez-Carrozzi study,^21^ a large overlap in the spectra of downstream target genes, including *CCL5*, *CCL2*, and *IL-6*, was found, thus indicating the broad relevance of these cellular mechanisms in homeostasis and disease.

The impact of the *CDK2AP1* loss or downregulation on the TME and, in particular, on the recruitment of PBMCs and the differentiation and polarization of infiltrating monocytes towards macrophages, was demonstrated both in vitro and in vivo. The secretome of *CDK2AP1*-KO tumor cells exhibits an extensive chemoattractant profile for monocytes and underlies the polarization of macrophages toward an M2-like state. By taking advantage of a multiplex immunofluorescence assay using tissue microarrays from a unique cohort of patient-derived OSCCs, we validated the correlation between CDK2AP1 downregulation and the increased presence of CD68^+^ macrophages. However, when it comes to tumor-associated macrophages with an M2-like profile, the overall differences with *CDK2AP1*-proficient tumors were not statistically significant, although high infiltration was only observed in *CDK2AP1*-low/negative tumors. This can most likely be attributed to technical limitations when attempting to distinguish M2-like macrophages from potential artifacts by solely relying on a combination of CD68 and CD163 markers. While a growing body of evidence supports the adverse prognostic role of CD68^+^ macrophages in many cancer types,^55-57^ the identification of different macrophage subpopulations in human tissues remains controversial^57-59^ because of the intrinsic limitations of surface marker-based analyses. While the latter are commonly used to infer functional phenotypes, the intricate nature of macrophage diversity and plasticity, influenced by complex epigenetic mechanisms as shown here, complicates this assumption.^60^

Recently, 2 interesting reviews pointed out how novel single-cell technologies, integrated with machine learning and artificial intelligence-based analyses, will help solve this issue.^60^^,^^61^ Historically, macrophages have been broadly classified as inflammatory (M1-like) or anti-inflammatory (M2-like) based on their distinct functions and specific markers identified through in vivo and in vitro assays. However, this classification has been challenged by the discovery of mixed expression patterns of M1-like and M2-like genes in human tumor-associated macrophages (TAMs) across various cancer types.^61^^,^^62^ The advent of single-cell RNA sequencing (scRNA-seq) has revolutionized the field by enabling the detailed molecular characterization of TAMs.^55^^,^^57^^,^^63^ These studies identified novel subsets of macrophages within tumors, taking advantage of the highest-expressing genes within scRNA-seq clusters for their classification. However, there remains a lack of consensus in naming the highest-expressing transcripts among different studies, resulting in some confusion in the definition of macrophage subtypes solely based on highly upregulated gene subsets. Recent advancements have shown that macrophages comprise multiple molecular subtypes and harbor diverse functions influenced by their spatial localization within the tumor microenvironment.^64^^,^^65^ This spatial distribution is crucial in determining macrophage function, as different regions within the TME can induce specific signaling events that shape macrophage activation and polarization. For instance, the hypoxic core of tumors can trigger the development of angiogenic TAMs through VEGF and ANG2 secretion.^66^

Furthermore, recent studies have shown that not only the tumor cell profile but also intra-tumoral lymphocytes can affect the nature of TAMs.^67^^,^^68^ CD8+ and CD4+ lymphocytes interact with both tumor cells and macrophages and determine the phenotype and the pro- or anti-tumor activity of the latter. Moreover, effective immunotherapy by checkpoint inhibition appears to associate with the induction of an M1-like TAM profile.^68^

Currently, the field is experiencing a transition from traditional M1/M2 classification to the molecular characterization of TAMs by means of more advanced techniques such as scRNA-seq and spatial transcriptomics. These advancements have paved the way for more precise identification and targeting of diverse functional subsets of macrophages within the TME, potentially leading to improved therapeutic strategies.^61^

In the near future, the implementation of single-cell multiome platforms will likely shed more light on these aspects of the TME biology. A recent spatial transcriptomic analysis of a small cohort of OSCC samples^69^ revealed distinct profiles between the tumor core (TC) and the leading edge (LE). Notably, while the expression profile characteristics of the invasive front revealed some degree of consistency across various cancer types, the equivalent profiles from the tumor cores exhibited tissue-specific characteristics. Evaluation of the prognostic impact of TC- and LE-specific gene signatures showed that high LE scores were consistently associated with poor prognosis and shortened disease-free survival (DFS) across multiple solid tumor types, including colorectal cancer, melanoma, and cutaneous squamous cell carcinoma.^69^ Analysis of CDK2AP1 protein expression in these tumor compartments could offer a unique perspective on how the competition between the NuRD and SWI/SNF complexes primes both the tumor cell and its surrounding microenvironment, thus favoring local and systemic dissemination.

Overall, our results show how epigenetic imbalance can affect autocrine and paracrine signaling in oral cancer cells and their direct environment, and elicit increased aggressiveness through secretion of pro-metastatic inflammatory molecules. This dual effect promotes cancer development while concurrently attracting PBMCs to the tumor and inducing the polarization of infiltrating monocytes into tumor-associated macrophages via inflammatory cytokines and chemokines.^41^

## Supplementary Material

djaf065_Supplementary_Data
