# Supplementary material for: The nucleosome remodeling and deacetylase-SWItch/sucrose non-fermentable antagonism regulates the coordinated activation of epithelial-to-mesenchymal transition and inflammation in oral cancer
Source: J Natl Cancer Inst. 2025 Mar 20;117(7):1438–55. doi: 10.1093/jnci/djaf065 (PMC12229464; doi:10.1093/jnci/djaf065)
Supplement: djaf065_Supplementary_Data [file djaf065_supplementary_data.zip › djaf065_Supplementary_Data/20241027_Sup_Figure_2.pdf]

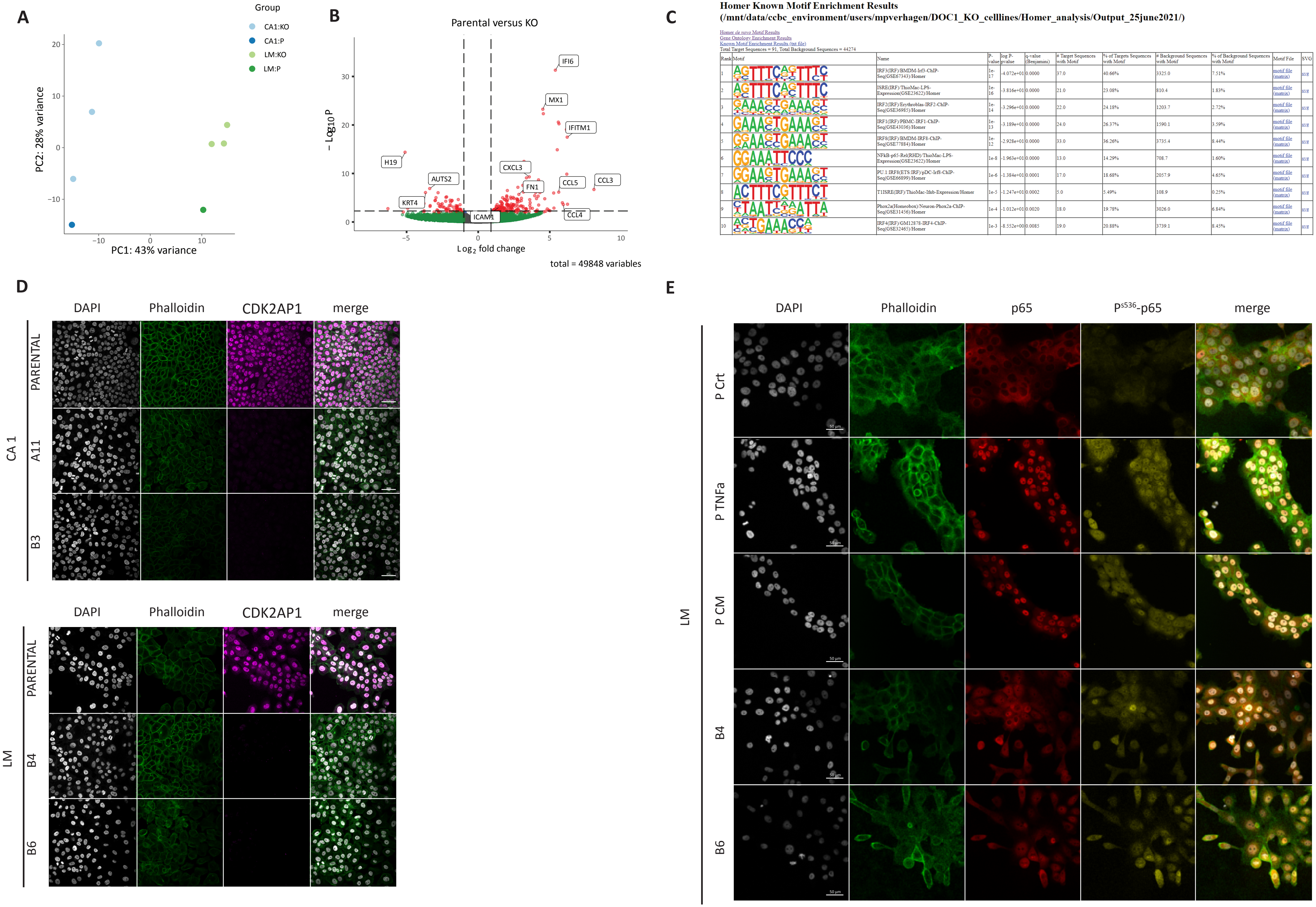

Homer Known Motif Enrichment Results (/mnt/data/ccbc_environment/users/mpverhagen/DOC1_KO_celllines/Homer_analysis/Output_25june2021/)

Homer *de novo* Motif Results
Gene Ontology Enrichment Results
Known Motif Enrichment Results (txt file)
Total Target Sequences = 91, Total Background Sequences = 44274

Rank	Motif	Name	P-value	log P-pvalue	q-value (Benjamini)	# Target Sequences with Motif	% of Targets Sequences with Motif	# Background Sequences with Motif	% of Background Sequences with Motif	Motif File	SVG
1	AGTTTCAGTTTC	IRF3(IRF)/BMDM-Irf3-ChIP-Seq(GSE67343)/Homer	1e-17	-4.072e+01	0.0000	37.0	40.66%	3325.0	7.51%	motif file (matrix)	svg
2	AGTTTCAGTTTC	ISRE(IRF)/ThioMac-LPS-Expression(GSE23622)/Homer	1e-16	-3.816e+01	0.0000	21.0	23.08%	810.4	1.83%	motif file (matrix)	svg
3	GAAAGTGAAAGT	IRF2(IRF)/Erythroblas-IRF2-ChIP-Seq(GSE36985)/Homer	1e-14	-3.296e+01	0.0000	22.0	24.18%	1203.7	2.72%	motif file (matrix)	svg
4	GAAAGTGAAAGT	IRF1(IRF)/PBMC-IRF1-ChIP-Seq(GSE43036)/Homer	1e-13	-3.189e+01	0.0000	24.0	26.37%	1590.1	3.59%	motif file (matrix)	svg
5	GGAAGTGAAAGT	IRF8(IRF)/BMDM-IRF8-ChIP-Seq(GSE77884)/Homer	1e-12	-2.928e+01	0.0000	33.0	36.26%	3735.4	8.44%	motif file (matrix)	svg
6	GGAAATTCCC	NFkB-p65-Rel(RHD)/ThioMac-LPS-Expression(GSE23622)/Homer	1e-8	-1.963e+01	0.0000	13.0	14.29%	708.7	1.60%	motif file (matrix)	svg
7	GGAAGTGAAAGT	PU.1:IRF8(ETS:IRF)/pDC-Irf8-ChIP-Seq(GSE66899)/Homer	1e-6	-1.384e+01	0.0001	17.0	18.68%	2057.9	4.65%	motif file (matrix)	svg
8	ACTTTCGTTTCT	T1ISRE(IRF)/ThioMac-Ifnb-Expression/Homer	1e-5	-1.247e+01	0.0002	5.0	5.49%	108.9	0.25%	motif file (matrix)	svg
9	CTAATTAAATTA	Phox2a(Homeobox)/Neuron-Phox2a-ChIP-Seq(GSE31456)/Homer	1e-4	-1.012e+01	0.0020	18.0	19.78%	3026.0	6.84%	motif file (matrix)	svg
10	ACTGAAACCA	IRF4(IRF)/GM12878-IRF4-ChIP-Seq(GSE32465)/Homer	1e-3	-8.552e+00	0.0085	19.0	20.88%	3739.1	8.45%	motif file (matrix)	svg
