# Supplementary material for: The nucleosome remodeling and deacetylase-SWItch/sucrose non-fermentable antagonism regulates the coordinated activation of epithelial-to-mesenchymal transition and inflammation in oral cancer
Source: J Natl Cancer Inst. 2025 Mar 20;117(7):1438–55. doi: 10.1093/jnci/djaf065 (PMC12229464; doi:10.1093/jnci/djaf065)
Supplement: djaf065_Supplementary_Data [file djaf065_supplementary_data.zip › djaf065_Supplementary_Data/Stabile_et al_JNCI_Supplementary materials and methods.docx]

**SUPPLEMENTARY MATERIALS AND METHODS**

***The NuRD-SWI/SNF antagonism regulates the coordinated activation of EMT and inflammation in oral cancer***

Roberto Stabile^1^, PhD; Francesco A. Tucci^1#*^, MD; Mathijs P. Verhagen^1*^, PhD; Carmen Embregts^2*^, PhD; Thierry P.P. van den Bosch^1^, PhD; Rosalie Joosten^1^, Ing; Maria J. De Herdt^3^, PhD; Berdine van der Steen^3^, Ing; Alex L. Nigg^1^, Ing; Senada Koljenović^1$^,MD, PhD; Jose A. Hardillo^3^, MD, PhD; C. Peter Verrijzer^4^, PhD; Adrian Biddle^5^, PhD; Robert J. Baatenburg de Jong^3^,MD, PhD; Pieter J.M. Leenen^6^, PhD and Riccardo Fodde^1§^, PhD.

^1^Department of Pathology, ^2^Viroscience, ^3^Otorhinolaryngology and Head & Neck Surgery, ^4^Biochemistry, and ^6^Immunology, Erasmus University Medical Center, Rotterdam, The Netherlands. ^5^Centre for Cell Biology and Cutaneous Research, Blizard Institute, Queen Mary University of London, UK.

*equal contributions

^§^to whom correspondence should be addressed at [r.fodde@erasmusmc.nl](mailto:r.fodde@erasmusmc.nl)

^#^current address: European Institute of Oncology IRCCS, Via Ripamonti 435, 20141 Milan, Italy.

^$^current address: Department of Pathology, Antwerp University Hospital, 2650 Edegem, Belgium

**Supplementary materials and methods**

*RT-qPCR* primers:

| **Gene** | **Forward primer** | **Reverse primer** |
| --- | --- | --- |
| *GAPDH* | 5′-ACCCAGAAGACTGTGGATGG-3′ | 5′-TCTAGACGGCAGGTCAGGTC-3′ |
| *CDK2AP1* | 5’-GGCAACGTCTTCACAGTACC-3’ | 5’-CCAGTCCTCTAGCGTGAATG-3’ |
| *FN1* | 5′-CAGTGGGAGACCTCGAGAAG-3′ | 5′-TCCCTCGGAACATCAGAAAC-3′ |
| *TWIST1* | 5′-GTCCGCAGTCTTACGAGGAG-3′ | 5′-GCTTGAGGGTCTGAATCTTGCT-3′ |
| *SLUG* | 5′-GGGGAGAAGCCTTTTTCTTG-3′ | 5′-TCCTCATGTTTGTGCAGGAG-3′ |
| *VIMENTIN* | 5′-GAGAACTTTGCCGTTGAAGC-3′ | 5′-GCTTCCTGTAGGTGGCAATC-3 |
| *N-CAD* | 5′-CAACTTGCCAGAAAACTCCAGG-3′ | 5′-ATGAAACCGGGCTATCTGCTC-3′ |
| *IL-6* | 5′-CACACAGACAGCCACTCACC-3′ | 5′-TTTTCTGCCAGTGCCTCTT-3′ |
| *IL-8* | 5′-TTGGCAGCCTTCCTGATTTC-3′ | 5′-TCTTTAGCACTCCTTGGCAAAAC-3′ |
| *NFKB1* | 5′-GTGGTGCCTCACTGCTAACT-3′ | 5′-GGATGCACTTCAGCTTCTGT-3′ |
| *NFKB2* | 5′-TAGCCACAGAGATGGAGGAG-3′ | 5′-CCGAGTCGCTATCAGAGGTA-3′ |
| *ICAM1* | 5′-CTGCAGACAGTGACCATC-3′ | 5′-GTCCAGTTTCCCGGACAA -3′ |
| *CCND1* | 5’-CCGTCCATGCGGAAGATC-3’ | 5’-CCTCCTCCTCGCACTTCTGT-3’ |
| *RELA* | 5’-GCACAGATACCACCAAGACC-3’ | 5’-TCAGCCTCATAGAAGCCATC-3’ |
| *RELB* | 5’-CATTGAGCGGAAGATTCAAC-3’ | 5’-GCAGCTCTGATGTGTTTGTG-3’ |

*TME multiplex immunofluorescence antibodies specifications:*

| **Antibody** | **Dilution** | **Species** | **Company** | **Clone** | **Incubation time** |
| --- | --- | --- | --- | --- | --- |
| CD3 | 2,05 ug/ml | Rabbit | Ventana | 2GV6 | 32 min. |
| CD14 | 1,04 ug/ml | Rabbit | Atlas Antibodies | polyclonal | 32 min. |
| CD163 | 2,14 ug/ml | Mouse | Cell Marque | MRQ-26 | 32 min. |
| CD68 | 3,49 ug/ml | Mouse | Ventana | KP1 | 20 min. |
| 34BE12/Cytokeratin | 4,05 ug/ml | Mouse | Cell Marque | 34BE12 | 4 min. |
