# Supplementary material for: The nucleosome remodeling and deacetylase-SWItch/sucrose non-fermentable antagonism regulates the coordinated activation of epithelial-to-mesenchymal transition and inflammation in oral cancer
Source: J Natl Cancer Inst. 2025 Mar 20;117(7):1438–55. doi: 10.1093/jnci/djaf065 (PMC12229464; doi:10.1093/jnci/djaf065)
Supplement: djaf065_Supplementary_Data [file djaf065_supplementary_data.zip › djaf065_Supplementary_Data/Supplementary Figure 3.pdf]

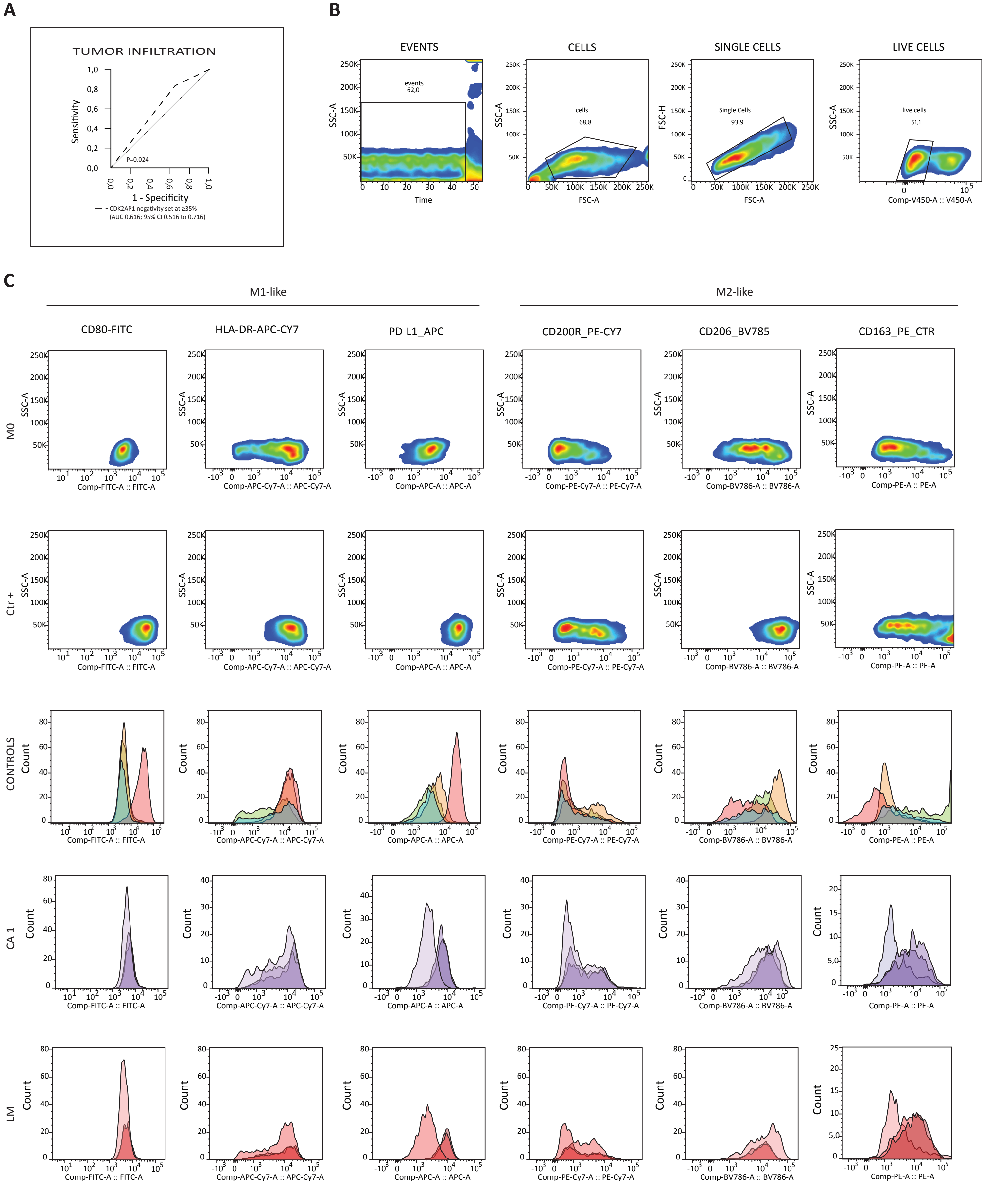

	Sample Name	Subset Name
	M0	live cells
	M1	live cells
	M2a	live cells
	M2c	live cells

	Sample Name	Subset Name
	CA1 P	live cells
	CA1 A11	live cells
	CA1 B3	live cells

	Sample Name	Subset Name
	LM P	live cells
	LM B4	live cells
	LM B6	live cells
