# Supplementary material for: The nucleosome remodeling and deacetylase-SWItch/sucrose non-fermentable antagonism regulates the coordinated activation of epithelial-to-mesenchymal transition and inflammation in oral cancer
Source: J Natl Cancer Inst. 2025 Mar 20;117(7):1438–55. doi: 10.1093/jnci/djaf065 (PMC12229464; doi:10.1093/jnci/djaf065)
Supplement: djaf065_Supplementary_Data [file djaf065_supplementary_data.zip › djaf065_Supplementary_Data/Supplementary Figure Legends.docx]

**Supplementary Figure 1. *CDK2AP1-KO confers epithelial mesenchymal plasticity in OSCC cell lines.***

1. Flow cytometric analysis of CA1 parental and *CDK2AP1*-KO cell lines (CA1-A11 and -B3) with antibodies directed against E-CAD and N-CAD. The E-CAD/N-CAD-low and -high regions were defined using the parental cell line as a reference.
2. *CDK2AP1* RT-qPCR analysis of shRNA induction in the parental CA1 and LM cell lines. Experiments were performed in triplicate, and mRNA expression was normalized to that of GAPDH. p-values denote one-way ANOVA and one-sample t-tests against uninduced parental cell lines (*p<0.05, **p<0.01, ***p<0.001).
3. Analysis of the maximal distance covered by the parental and *CDK2AP1*-KO CA1 and LM cell lines in the invasion assay ( lower panel in Fig. 1C). Each dot represents a single cell measurement. Experiments were performed in triplicate, and p-values denote one-way ANOVA and one-sample t-tests against the parental cell lines (*p<0.05; **p<0.01; ***p<0.001).
4. IHC analysis of tumors obtained by subcutaneous injection of either CA1 parental or *CDK2AP1*-KO clones (CA1-A11 and -B3) with antibodies against human mitochondria and FN1. In the left column, lower magnification (4X) images show a specific area, which is shown at higher magnification (20X) in the right column. Lower magnification scale bars: 500 μm; higher magnification field scale bars: 250 μm.

**Supplementary Figure 2. *RNAseq analysis of parental and CDK2AP1-KO OSCC cancer cell lines reveals the downstreamregulation of the inflammatory pathways.***

1. Principal component analysis (PCA) of RNA-seq profiles from parental and *CDK2AP1*-KO OSCC cell lines.
2. Volcano plots of differentially expressed genes upon *CDK2AP1* ablation in our panel of OSCC cell lines (log_2_ fold change> 2). On the right side of the plot, genes upregulated upon deletion of CDK2AP1 in CA1 and LM-KO clones are shown, whereas on the left side, the genes downregulated after CDK2AP1 KO are represented.
3. HOMER transcription factor motive analysis of differentially expressed genes upon CDK2AP1 ablation in CA1 and LM OSCC cell lines.
4. Representative immunofluorescence images of CDK2AP1 protein expression in parental and *CDK2AP1*-KO cell lines. The upper panel shows CA1 parental cells and KO clones A11 and B3, while the lower panel depicts LM parental cells and KO clones B4 and B6. Cells were fixed with 4% paraformaldehyde and stained with antibodies against CDK2AP1 (magenta). Nuclei were visualized using DAPI (gray), and actin filaments with phalloidin (green).
5. Representative immunofluorescence images of p65 and PS536-p65 in LM parental and CDK2AP1-KO cell cultures under normal conditions (Ctr/B4/B6), following treatment with 10 ng/ml TNF-α (TNFa), or with conditioned medium (CM) from CDK2AP1-KO clones for 1 hour. Cells were fixed with 4% paraformaldehyde and stained with antibodies against p65 (red) or phosphorylated PS536-p65 (yellow). Nuclei were stained with DAPI (gray), and actin filaments with phalloidin (green). Scale bar: 50 µm.

**Supplementary Figure 3. *CDK2AP1 status influences the immune cells of the tumor microenvironment (TME).***

1. The ROC (receiver operating characteristic) curve, based on which the optimal threshold of 35.0% of CDK2AP1-negative cells was established and employed for tumor infiltration analysis.
2. Cytofluorimetric pre-processing strategy for the detection of different states of macrophage polarization. Briefly, the number of cells was determined based on the cell size over time (EVENTS). Next, using FSC-A and FCS-H, the cells were first separated by debris (CELLS), and then SINGLE CELLS were purified (SINGLE CELLS). Eventually, thanks to ZombieViolet staining, we focused on living cells (LIVE CELLS) (see also Materials and Methods).
3. Expression of macrophage polarization markers by cytometric bead assays. The first lane shows the profile of each marker detected in our negative control sample (M0 macrophages) while the second lane shows the profile of the positive controls (Ctr +): for the M1-like specific markers, the M1-like macrophages treated with INF-γ + LPS are shown; for M2-like macrophages, the ones treated with IL-4 and IL-10 are reported. The third lane shows the different florescence levels of the controls taken in the exams per marker (CONTROLS). The last two lanes show the different profiles obtained when the macrophages were stimulated with the supernatant of the CA1 and LM cell lines, respectively (parental profiles in light colors; CDK2AP1 KO clones in dark colors).
