# Supplementary figures and images for: The nucleosome remodeling and deacetylase-SWItch/sucrose non-fermentable antagonism regulates the coordinated activation of epithelial-to-mesenchymal transition and inflammation in oral cancer
Source: J Natl Cancer Inst. 2025 Mar 20;117(7):1438–55. doi: 10.1093/jnci/djaf065 (PMC12229464; doi:10.1093/jnci/djaf065)

A

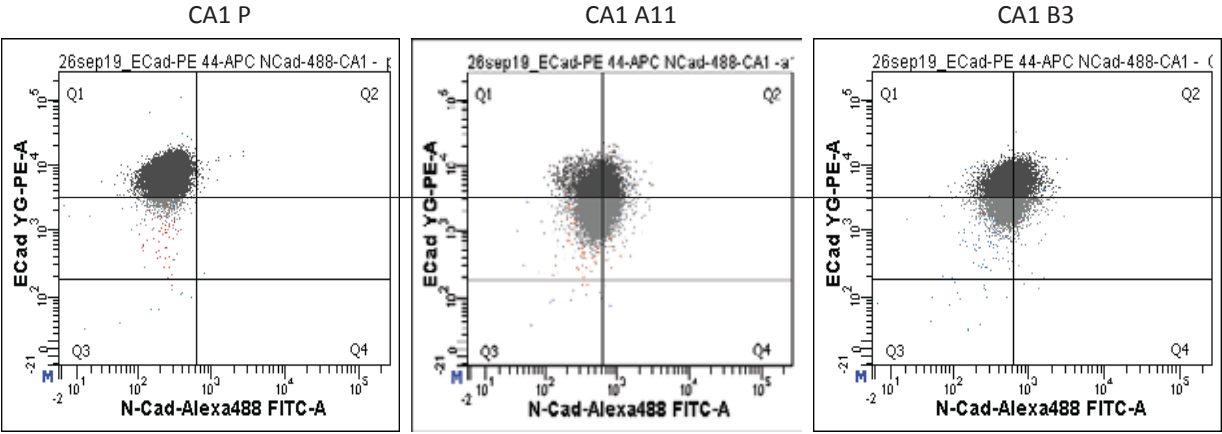

B

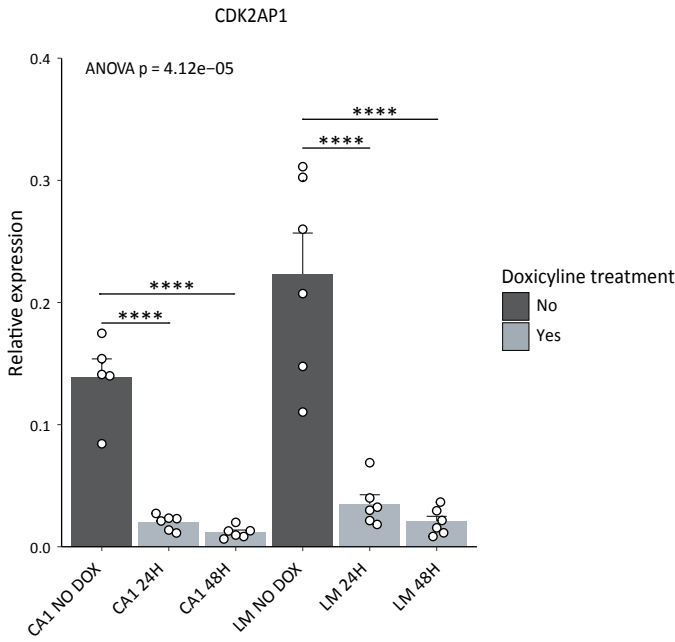

C

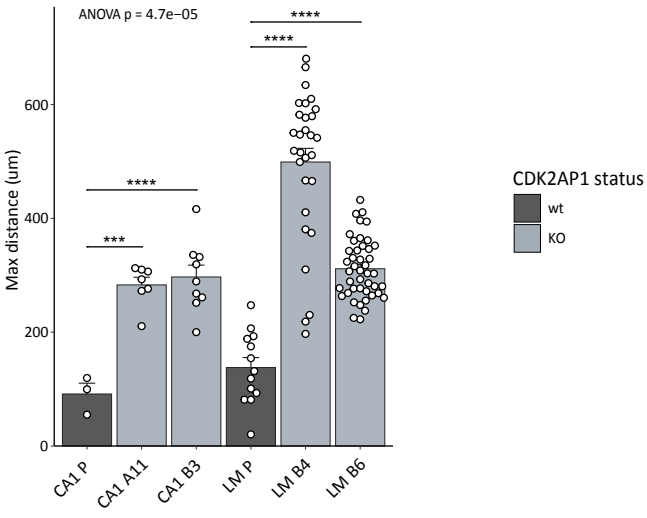

D

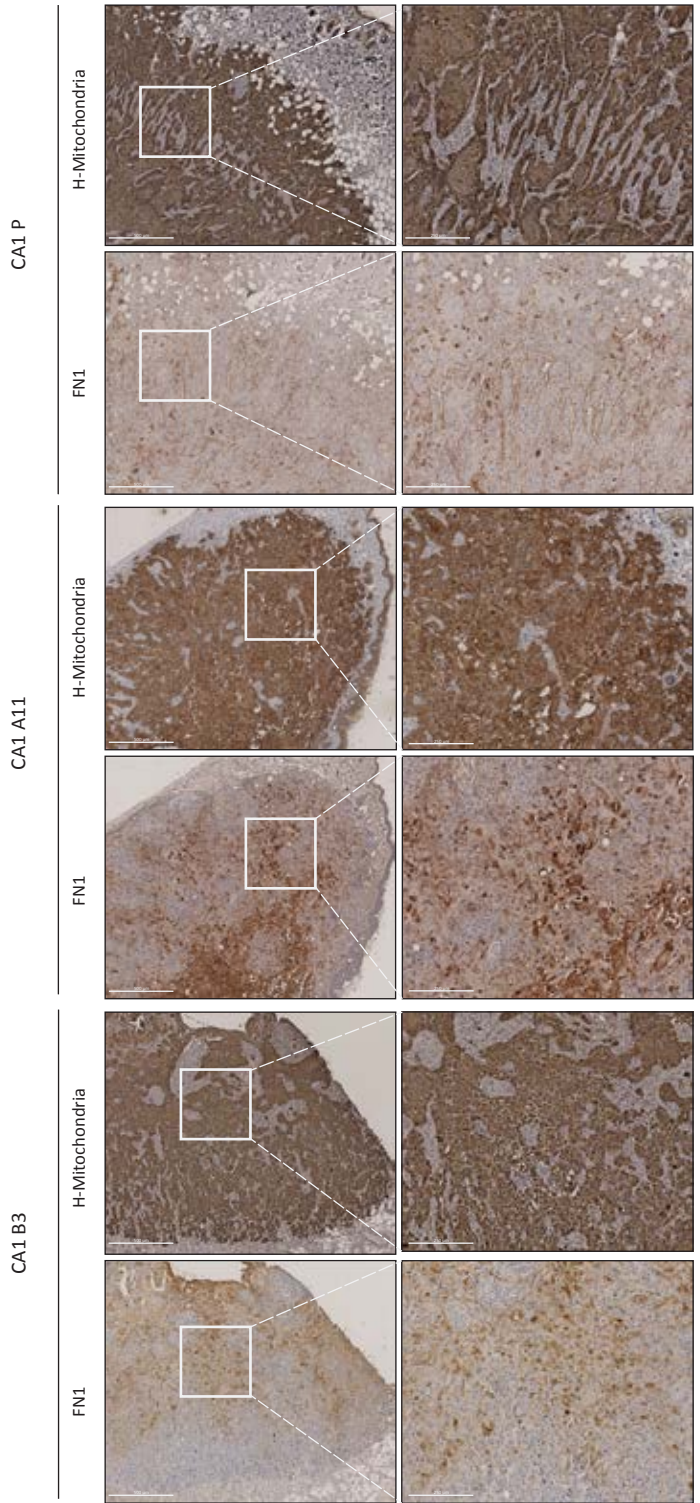

Supplement: djaf065_Supplementary_Data [file djaf065_supplementary_data.zip › djaf065_Supplementary_Data/Supplementary Figure 1_compressed.pdf]
